# Exosome crosstalk between cancer stem cells and tumor microenvironment: cancer progression and therapeutic strategies

**DOI:** 10.1186/s13287-024-04061-z

**Published:** 2024-11-22

**Authors:** Qi Li, Guangpeng He, Yifan Yu, Xinyu Li, Xueqiang Peng, Liang Yang

**Affiliations:** Department of General Surgery, The Fourth Affiliated Hospital, China Medical University, Shenyang, 110032 China

**Keywords:** Cancer stem cells, Exosomes, Extracellular vesicles, Tumor microenvironment, Cancer treatment

## Abstract

Cancer stem cells (CSCs) represent a small yet pivotal subset of tumor cells endowed with self-renewal capabilities. These cells are intricately linked to tumor progression and are central to drug resistance, metastasis, and recurrence. The tumor microenvironment (TME) encompasses the cancer cells and their surrounding milieu, including immune and inflammatory cells, cancer-associated fibroblasts, adjacent stromal tissues, tumor vasculature, and a variety of cytokines and chemokines. Within the TME, cells such as immune and inflammatory cells, endothelial cells, adipocytes, and fibroblasts release growth factors, cytokines, chemokines, and exosomes, which can either sustain or disrupt CSCs, thereby influencing tumor progression. Conversely, CSCs can also secrete cytokines, chemokines, and exosomes, affecting various components of the TME. Exosomes, a subset of extracellular vesicles (EVs), carry a complex cargo of nucleic acids, proteins, and lipids, playing a crucial role in the communication between CSCs and the TME. This review primarily focuses on the impact of exosomes secreted by CSCs (CSC-exo) on tumor progression, including their roles in maintaining stemness, promoting angiogenesis, facilitating metastasis, inducing immune suppression, and contributing to drug resistance. Additionally, we discuss how exosomes secreted by different cells within the TME affect CSCs. Finally, we explore the potential of utilizing exosomes to mitigate the detrimental effects of CSCs or to target and eliminate them. A thorough understanding of the exosome-mediated crosstalk between CSCs and the TME could provide valuable insights for developing targeted therapies against CSCs.

## Introduction

Cancer remains the leading cause of death worldwide and is a major impediment to increasing human lifespan across all nations globally [1]. A recent statistics indicate approximately 20 million new cancer cases and about 9.7 million deaths attributed to the disease in 2020 [2]. At present, there are several therapeutic options available for cancer, such as surgery, radiotherapy, chemotherapy, targeted therapy, and immunotherapy. However, issues like tumor recurrence, metastasis, and resistance to drugs continue to pose significant challenges in effective cancer management [3]. A key reason for these challenges is that most treatments predominantly target bulk tumor cells and do not effectively address cancer stem cells (CSCs) [4]. CSCs were first discovered and isolated in leukemia in 1994 [5]. These cells share many characteristics with stem cells, including self-renewal and the ability to proliferate indefinitely. CSCs are distinguished by unique stem cell-like properties and express specific markers that differ from those of other tumor cells. These markers vary across different types of cancer, allowing for the relatively specific isolation of CSCs. Since their discovery in leukemia, CSCs have been identified and isolated from various other tumors, including those affecting the esophagus, pancreas, ovaries, colon, liver, breast, prostate, and skin, among others. Many scientists widely recognize the pivotal role of CSCs in tumor progression due to their capacity for self-renewal and unlimited proliferation. CSCs also possess robust migratory and invasive capabilities, enabling them to travel to distant sites through the bloodstream or lymphatic system and establish metastases. Furthermore, their resistance to conventional therapies means that any surviving CSCs can regenerate the tumor, leading to treatment failures and recurrence. CSCs also demonstrate “plasticity,” which allows them to differentiate into various cell states and adapt to environmental changes, thereby enhancing tumor progression and contributing to increased drug resistance [6, 7]. Currently, there are two models explaining tumor cell heterogeneity. The first is the hierarchical organization theory, which suggests that a structured hierarchy exists within tumors, with cancer stem cells at the apex. These cells are characterized by their unique ability to self-renew and differentiate into various cell types found within the tumor. The CSC model posits that certain cancer cell populations possess self-renewal capabilities that can initiate tumor growth, and these cells generally exhibit lower levels of differentiation compared to most cancer cells [8]. The second model related to CSCs is the concept of clonal evolution. This model proposes that tumors evolve within the host’s biological environment through mutations and natural selection. Within this framework, CSCs are considered clonal populations with specific mutations that confer stem-like properties, such as the ability to self-renew and resist treatments. This concept emphasizes the genetic heterogeneity and adaptability of tumors, highlighting the complexities of targeting CSCs for cancer therapy [7, 9]. As a result, CSCs are often seen as the primary drivers behind tumor growth and pose significant obstacles in cancer therapy.

The tumor microenvironment (TME) encompasses not only the cancer cells but also the surrounding environment, which is made up of a variety of elements such as immune and inflammatory cells, carcinoma-associated fibroblasts, nearby stromal tissue, tumor blood vessels, and a range of cytokines and chemokines [10, 11]. CSCs are intricately connected to their environment, engaging in continuous interactions. They can alter the TME by releasing signaling molecules, enhancing angiogenesis, promoting immune tolerance, and aiding metastasis. Conversely, cells within the TME, along with their secreted factors, can bolster the self-renewal and stemness of CSCs, stimulate angiogenesis, attract immune and stromal cells, and facilitate tumor invasion and metastasis. Extracellular vesicles (EVs) play a pivotal role in facilitating communication between CSCs and TME cells.

**Fig. 1:**
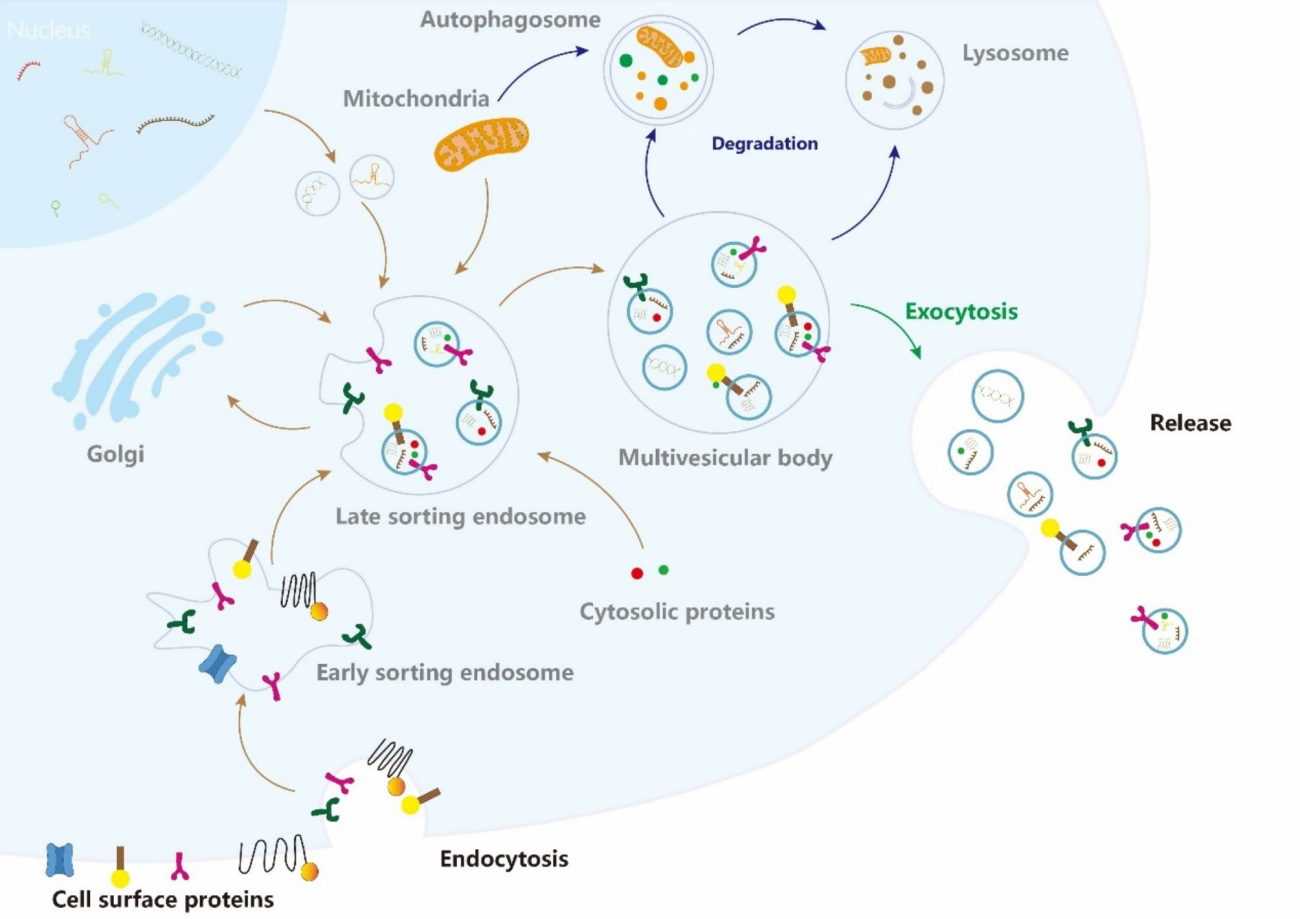
Exosomes biogenesis and release. Exosome formation and release encompass several steps, starting with endocytosis, followed by the formation of multivesicular bodies (MVBs), and culminating in the release of exosomes into the extracellular space via fusion with the plasma membrane

EVs are lipid bilayer-enclosed particles released by all cell types. They contain cargo from their donor cells but lack functional nuclei and cannot replicate. Found in various biological fluids such as blood, urine, semen, saliva, breast milk, cerebrospinal fluid, and bile. EVs are considered a mechanism for cells to eliminate harmful or redundant intracellular components. They transport a variety of substances, including DNA, RNA, lipids, and proteins, enabling them to transmit signals and effect physiological changes in recipient cells, thereby serving as crucial conduits for intercellular communication. The powerful cargo-carrying capacity of EVs makes them crucial in the interaction between CSCs and cells within TME. Exosomes, a type of EVs, are produced through a two-step invagination process of the cytoplasmic membrane, resulting in the formation of multivesicular bodies (MVBs) containing intraluminal vesicles (ILVs). These ILVs are eventually released as exosomes, which are approximately 40 to 160 nm in diameter. MVBs fuse with the cytoplasmic membrane and release exosomes into the extracellular space via exocytosis [12, 13](Fig. 1). These exosomes carry host cell-specific cargo from their host cell and are internalized by recipient cells via endocytosis, releasing their contents and promoting intercellular communication. This mechanism is crucial for interactions between CSCs and TME cells. Exosomes transport bioactive molecules like proteins, lipids, and RNAs, which regulate the behavior of both CSCs and the surrounding stromal cells. This bidirectional communication not only supports tumor growth, angiogenesis, and immune evasion but also plays a role in tumor progression. For instance, exosomes secreted by CSCs (CSC-exo) can transform normal stromal cells into a state conducive to tumorigenesis, thereby supporting the survival and proliferation of CSCs. Moreover, exosomes within the TME can affect the plasticity and resistance to therapy of CSCs. Given the focus of current research on EV-mediated communication in the TME mainly focuses on exosomes, this review will specifically highlight the role of exosomes.

## The role of CSC-exo in the TME

### CSC-exo promotes stemness in Non-CSCs

Currently, substantial evidence suggests that CSCs can impart stemness traits from parental cells to non-CSCs via exosomes, thus increasing tumor stemness and accelerating tumor progression. Researches are finding that CSC-exo contain proteins that trigger stemness signaling pathways in non-CSCs, like Wnt, Notch, and Hedgehog, to regulate tumor stemness. For instance, exosomes produced by glioblastoma stem cells (GSCs) function as carriers, transporting Notch1 protein to non-CSCs. This transfer activates the Notch1 signaling pathway, inducing the differentiation of glioma cells into GSCs and thus enhancing tumor stemness and tumorigenic potential. When Notch1 RNA interference is applied, it decreases Notch1 protein levels in GSCs and their exosomes, subsequently reducing the levels of stemness-related proteins in the targeted non-GSCs [14]. Huang et al. discovered that the release of liver CSC-exo was Rab27A dependent, while Rab27A expression was closely related to Nanog in hepatocellular carcinoma (HCC) tissues. CSC-exo can elevate Nanog expression in non-CSCs, indicating that Rab27A contributes to the enhancement of non-CSC stemness in HCC by regulating the release of CSC-exo [15].

Additionally, numerous studies have found that RNA molecules encapsulated in CSC-exo can activate stemness-related pathways, conferring stemness to non-CSCs. Li and colleagues demonstrated that *FMR1-AS1*, a long non-coding RNA (lncRNA) found in exosomes from esophageal CSCs, binds to the endosomal toll-like receptor 7 (TLR7) and activates NFκB signaling. This activation promotes the expression of the stemness-related gene c-Myc in recipient non-CSCs, enhancing their malignant potential [16]. Similarly, colorectal CSC-exo and thyroid CSC-exo can carry miRNAs and lncRNAs that target cancer cells to activate the stemness-related Wnt-β-catenin signaling pathway, thereby conferring stemness to non-CSCs [17, 18]. Han et al. discovered that that circular RNAs (circRNAs), specifically *circ-ZEB1* and *circ-AFAP1*, in liver CSC-exo significantly elevate the expression of the stemness marker CD133, while simultaneously decreasing levels of E-cadherin and epithelial cell adhesion molecule (EpCAM), markers associated with the epithelial-mesenchymal transition (EMT) process. This activity not only boosts the stemness and EMT process in cancer cells but also advances the progression of HCC. Additionally, the levels of *circ-ZEB1* and *circ-AFAP1* are markedly elevated in HCC tissues compared to peritumoral tissue, and Their expression shows a positive correlation with the stemness marker CD133. Therefore, these two indicators can also serve as prognostic markers for HCC [19].

### CSC-exo promotes tumor angiogenesis

Tumor angiogenesis is a critical mechanism in tumor development, as it supplies the necessary oxygen and nutrients to tumor cells through blood vessels [20]. CSCs primarily promote angiogenesis by secreting pro-angiogenic factors and exosomes [21]. Some evidence suggests that CSC-exo contains numerous pro-angiogenic components and facilitates tumor angiogenesis by interacting with endothelial and stromal cells within the TME, activating key signaling pathways. For example, some researches have shown that CSCs can influence the differentiation of endothelial progenitor cells through the exosomal delivery of miRNAs [22]. Additionally, exosomal *miR-26a* secreted by GSCs enhances the angiogenic capability of human brain microvascular endothelial cells (ECs) by triggering the PI3K/Akt signaling pathway and reducing PTEN expression [23]. Interestingly, other study found that exosomes derived from GSCs (GSC-exo) are also rich in *miR-21* and VEGF, which enhance the angiogenic capability of ECs by activating the VEGF/VEGFR2 signaling pathway [24, 25]. Moreover, Lindoso and his colleagues discovered that renal CSC-exo can modify the secretion profiles of mesenchymal stem cells (MSCs), inducing them to release interleukin-8 (IL-8), osteopontin (OPN), and myeloperoxidase (MPO). This alteration enhances tumor vascularization and growth, significantly increasing tumor migration and stroma remodeling [26]. IL-8 can induce ECs to produce VEGF-A and raise VEGFR2 expression, as well as enhancing MMP secretion which breaks down the extracellular matrix, facilitating the proliferation of ECs to form new blood vessels [27]. Additionally, OPN is abundantly expressed in the tumor stroma and participates in the regulatory signaling processes associated with angiogenesis, metastasis and tumor growth across various cancers [28]. MPO plays a role in ECs activities linked to angiogenesis, promoting the expression of angiogenic signaling pathways and their associated genes [29].

### CSC-exo promotes tumor invasion, migration, and metastasis

CSCs are one of the root causes of metastatic spread [30], which may be mainly dependent on CSC-exo, and transfer CSC characteristics to non-CSCs. EMT plays a vital role in this process, marking a key transition in tumor invasion and metastasis. During EMT, epithelial cells lose their characteristic features and gain mesenchymal traits, including enhanced mobility and invasiveness [31]. This transformation involves the loss of intercellular adhesion and epithelial markers such as cytokeratins and E-cadherin, coupled with an increase in mesenchymal markers like N-cadherin, vimentin, and fibronectin [32]. Numerous factors termed EMT-inducing transcription factors (EMT-TFs), including ZEB1/2, SNAIL, SLUG, and TWIST, can stimulate EMT in cancer cells, thereby promoting tumor invasion [33]. CSC-exo can act as a signal carrier, transferring EMT signals to tumor cells, hence promoting tumor migration, invasion, and metastasis. In thyroid cancer, CSC-exo significantly upregulates *lncRNAs MALAT1* and *linc-ROR*, along with the EMT marker SLUG and the stem cell transcription factor SOX2. These molecules are transferred to non-CSCs, triggering EMT and thus enhancing tumor metastasis [34]. Multiple studies indicate that CSC-exo cannot only directly transfer EMT-TFs but also convey EMT signals via non-coding ncRNAs to promote EMT. Alzahrani et al. found significant levels of *miR21*, *lncTuc339*, *lncHEIH*, and HCC *lncHOTAIR* in liver cancer CSC-exo. These non-coding RNAs affect the mRNA levels of Bcl2, TGFβ1, NFκB, VEGF, and matrix metalloproteinase 9 (MMP9), thereby activating corresponding signaling pathways that significantly increase cancer cell metastasis and induce EMT [35]. Furthermore, Wu et al. discovered that *lncRNA CDKN2B-AS1* in CSC-exo can enhance tumor cell viability, invasion, and EMT was achieved by decreasing the levels of E-cadherin and increasing the levels of P4HA1, which is an enzyme responsible for collagen production and deposition, as well as N-cadherin and vimentin expression [36]. Wang and colleagues discovered that *miR-210-3p* in lung CSC-exo elevates the expression of N-cadherin, vimentin, MMP-9, and matrix metalloproteinase 1 (MMP-1) in lung cancer cells, while simultaneously decreasing E-cadherin expression to promote cell migration and invasion [37]. Additionally, *miR-19b-3p* in clear cell renal cell carcinoma CSC-exo augments the expression of EMT-related genes by targeting the PTEN signaling pathway. Research has also shown that integrin CD103, carried by CSC-exo, enables organ-specific metastasis to the lungs, highlighting the role of CSC-exo in directing tumor metastasis [38].

The non-coding RNAs transported by CSC-exo can directly trigger gene expression related to the EMT in cancer cells to promote EMT, and they also indirectly influence tumor metastasis through alternative pathways. Some studies have found that the suppression of the autophagy pathway by CSC-exo can promote the metastasis and colonization of tumors. For instance, *miR-4535* and *miR-1268a* in melanoma CSC-exo have been found to enhance the metastatic colonization of melanoma cells by inhibiting the autophagy pathway [39, 40]. Zhu et al. identified that the SOX2-β-catenin/Beclin1/autophagy signaling axis in colorectal CSCs plays a role in regulating chemoresistance, CSC traits, and EMT in colorectal cancer (CRC) [41]. SOX2, which is typically a marker of CSCs, is commonly released via exosomes [42]. Additional studies indicate that CSC-exo can indirectly boost tumor metastasis by increasing glycolysis. In lung CSC-exo, the highly expressed lncRNA *Mir100hg* targets *miR-15a-5p* and *miR-31-5p* in tumor cells, elevating glycolytic activity and thereby enhancing metastatic potential [43]. Ji et al. demonstrated that in liver cancer, exosomal *ZFPM2-AS1* modulates glycolysis in HCC via a pyruvate pathway dependent on HIF-1α, affecting tumor metastasis and growth, while also promoting macrophage recruitment and M2 polarization [44]. Additionally, CSC-exo can influence EMT by regulating immune cells. Hwang et al. found that colorectal CSC-exo enhances the activity of the neutrophils from bone marrow and promotes their pro-tumor characteristics. Moreover, patients with CRC displaying active CSC signaling (characterized by Snail + and IL8 + markers) showed elevated levels of MPO + tumor-infiltrating neutrophils, suggesting neutrophils are involved in mediating EMT and maintaining the CSC niche [45].

### CSC-exo promotes immunosuppression

The immune system performs three fundamental functions: immune defense, which combats pathogenic microorganisms and eradicates invading pathogens and harmful biomolecules; immune surveillance, which monitors and swiftly eliminates mutated cells; and immune stability, which ensures self-regulation by recognizing self, distinguishing between “foreign” or “harmful” entities, and maintaining overall homeostasis. Typically, immune cells actively execute these functions by recognizing, monitoring, and clearing malignant cells. However, these malignant cells often deploy sophisticated strategies to escape immune detection and destruction, with CSCs playing a pivotal role in this process. The mechanisms by which CSCs influence infiltrating immune cells are currently understood to be highly complex. Research indicates that CSC-exo can specifically target certain subsets of immune cells, such as tumor-associated macrophages (TAMs) and T cells [46].

CSC-exo can polarize TAMs into a pro-tumor MHC-II phenotype, thereby impacting tumor immunity [46]. In recent years, some research found MHC-II-macrophages play multiple roles in the TME, including immune suppression [47], angiogenesis [48], ECM deposition [49], and promoting metastasis [50, 51]. Gabrusiewicz and colleagues found that GSC-exo drives the differentiation of monocytes into M2 macrophages via the EIF2, mTOR, ephrinB signaling pathways, and STAT3 phosphorylation. This process enhances the expression of programmed death-ligand 1 (PD-L1), a key immune checkpoint extensively studied in clinical trials. PD-L1 binds to the PD-1 receptor on T cells, creating an immunosuppressive environment that facilitates tumor immune evasion [52, 53]. Furthermore, research has revealed that small EVs secreted by oral squamous cell carcinoma (OSCC) CSCs promote M2 macrophage polarization through the transfer of *lncRNA UCA1*. This lncRNA targets the LAMC2-mediated PI3K/AKT signaling axis and also inhibits the proliferation of CD4^+^ T cells and the production of interferon-gamma (IFN-γ) [54].

Additionally, some research has found that exosomes can influence tumor immunity by modulating the cytotoxic function of natural killer (NK) cells and T cells through TAMs [55]. Apart from these indirect effects through TAMs, CSC-exo directly influences T cells as well. For example, esophageal cancer CSC-exo can deliver O-GlcNAc transferase (OGT) to adjacent CD8^+^ T cells, increasing their PD-1 expression. This activation adversely affects the proliferation and functionality of CD8^+^ T cells, facilitating cancer immune evasion [56]. In CRC, CSC-exo increases TNF-α expression in neutrophils, and this upregulation, stimulated by IL-1β, may lead to activation-induced cell death of T cells, thus intensifying tumor immune suppression [45]. Another study found that colorectal CSC-exo containing *miRNA-146a-5p* promotes the stem-like properties and tumor-forming ability of CRC cells by targeting Numb [57]. Patients with elevated serum levels of exosomal *miRNA-146a-5p* show increased CSC characteristics, fewer tumor-infiltrating CD8^+^ T cells, and more tumor-infiltrating CD66b^+^ neutrophils, leading to tumor immune suppression. Further research indicates that brain CSC-exo contains the extracellular matrix protein tenascin-C (TNC), which interacts with integrin receptors α5β1 and αvβ6. This interaction suppresses T cell mTOR pathway signaling, reducing T cell activity in co-culture and allowing tumor cells to evade destruction by immune cells [58]. Moreover, Naseri et al. found that CSC-exo can increase the ratio of IL-12 to IL-10 in dendritic cells (DCs). They also discovered that CSC-exo contains immunogenic antigens that promote anti-tumor responses. DCs loaded with these CSC-exo can observably promote the growth of autologous T cells and activate cytotoxic T cells targeted at spheroids, enhancing in vivo anti-tumor effects [59]. While the focus has largely been on these specific immune cells, further studies may gradually uncover the direct effects of CSC-exo on other immune cells like mast cells and NK cells, which play significant roles in the body’s defense against tumors. Future studies may gradually uncover the connections between CSC-exo and these cells, potentially revealing more mechanisms of tumor immune suppression.

### CSC-exo promotes tumor drug resistance

Since cancer is often diagnosed at advanced stages, targeted therapies and chemotherapy are typically the most viable treatment options [60]. However, tumors frequently develop resistance to these treatments, posing a significant hurdle in cancer therapy. Thus, understanding the mechanisms underlying tumor drug resistance is crucial for improving treatment outcomes.

**Fig. 2:**
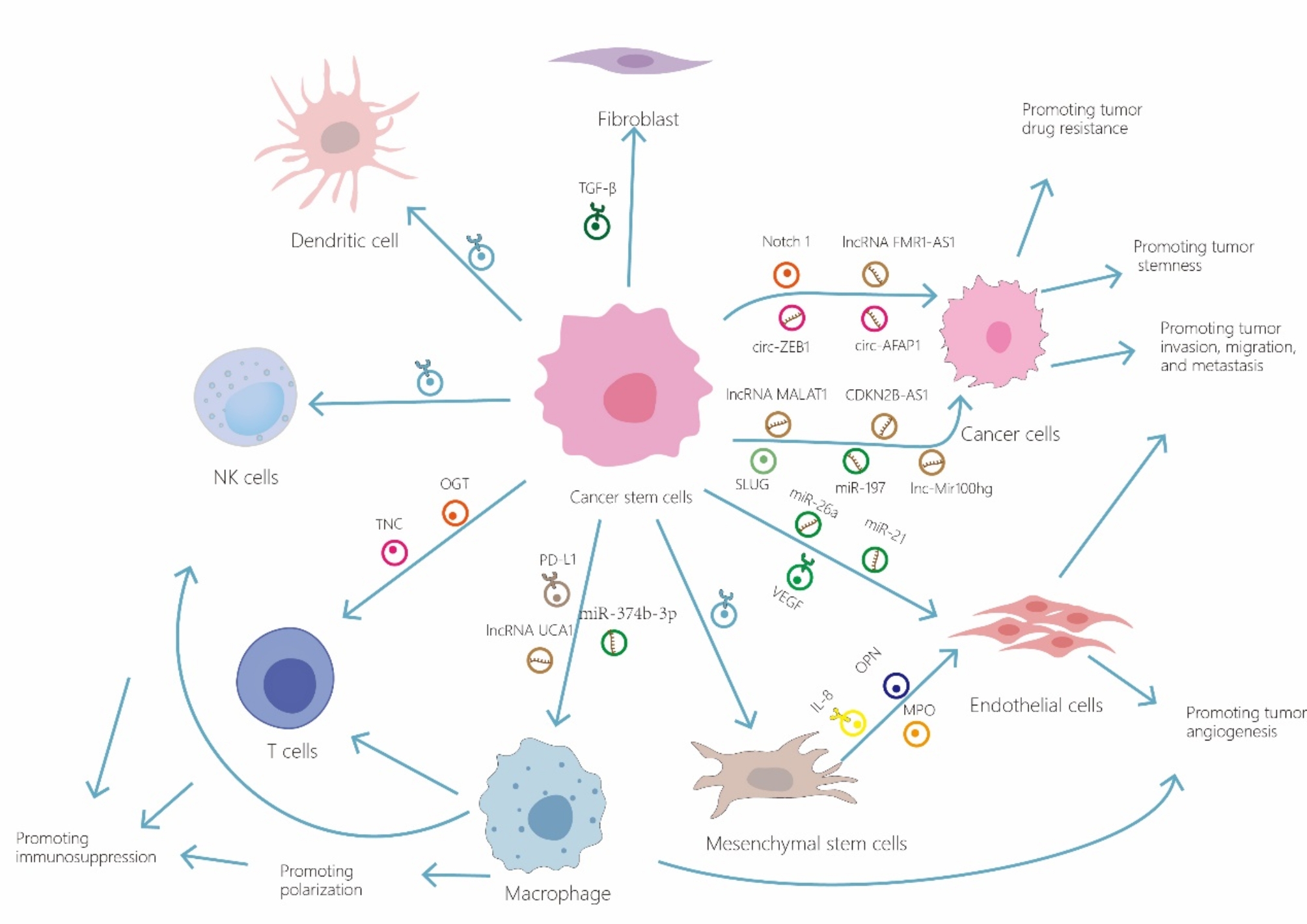
Cancer stem cell-derived exosomes are released into the cells within the tumor microenvironment, where they play a role in influencing tumor progression

Drug resistance is also a notable trait of CSCs. Studies have shown that CSC-exo can transport RNAs to adjacent non-CSCs, activating specific signaling pathways and imparting drug resistance to tumor cells. For instance, Yang and colleagues found that pancreatic CSC-exo resistant to gemcitabine can transfer *miR-210* to tumor cells, enhancing their drug resistance by upregulating resistance-associated proteins such as MDR1, YB-1, and BCRP. They noted that *miR-210* predominantly facilitates the horizontal transfer of resistance traits via the mTOR signaling pathway [61]. Santos and colleagues discovered that CSCs secrete exosomal *miRNA-155*, which can lead to the downregulation of C/EBP-β, thereby inhibiting the expression of TGF-β, C/EBP-β, and FOXO3a, resulting in the development of EMT and chemoresistance in cancer cells [62]. Yang and colleagues have found that GSC-derived EVs contain the *lncRNA MALAT1*, which influences immune effector cells in the central nervous system. This occurs through the *miR-129-5p*/HMGB1 axis, modulating lipopolysaccharide (LPS) response and promoting microglial polarization to an M2 phenotype. This polarization results in increased secretion of IL-6, IL-8, and TNF-α, impacting tumor resistance, growth, angiogenesis, and other facets of tumor progression [63]. Exosomes from breast CSCs also contribute to regulating tumor resistance by modulating autophagy. These exosomes deliver annexin-A6 (ANXA6), which influences the YAP1/Hippo pathway, enhancing autophagy and stemness in BC cells, thereby augmenting their resistance to paclitaxel (PTX) [64]. Similarly, Lu and Yao et al. confirmed that the YAP1 pathway influences autophagy, enhancing stemness and drug resistance in tumor cells [65, 66](Fig. 2) Table 1.

## Effects of exosomes derived from various sources within the TME on CSCs

### Effects of exosomes derived from cancer cells on CSCs

Non-CSC-derived EVs commonly transport specific cargo molecules that elevate the expression of stemness markers and activate various stemness-related signaling pathways in recipient CSCs. These pathways help maintain CSC characteristics, thereby driving tumor progression. Common stemness signaling pathways cover WNT/β-catenin [67], Notch [68], Hedgehog [69], and STAT pathways [70]. Studies have demonstrated that chemotherapy triggers cancer cells to release exosomes enriched with miRNAs associated with drug resistance. These miRNAs operate through stem cell pathways to augment CSC stemness and drug resistance. For example, chemotherapy triggers the EZH2/STAT3 pathway, leading to the secretion of exosomes from BC cells that harbor *miR-378a-3p* and *miR-378d*. These miRNAs target NUMB and DKK3, modulating the WNT/β-catenin and Notch pathways, improving CSC traits and drug resistance [71]. Additionally, exosomes released by endometrial cancer cells, containing Exosome component 5 (EXOSC5), can target netrin4 (NTN4) and bind to integrin β1, activating the FAK/SRC/β-catenin signaling axis and enhancing c-MYC activity, which in turn strengthens CSC properties and promotes tumor progression [72]. Additional studies suggest that non-CSC-derived EVs in bladder cancer carry ribosome-rich cargo that CSCs can absorb and use to rapidly initiate protein synthesis, boosting the expression of stemness factors such as ALDH1α1, OCT-4, TWIST, and CD44. Intriguingly, these studies have observed that while chemotherapy eliminates most cancer cells, these cells, prior to their demise, release EVs that support CSC survival in a chemotherapeutic environment, leading to drug resistance and disease progression [73]. In summary, exosomes derived from cancer cells play a pivotal role in the TME, profoundly affecting CSC behavior. They bolster CSC stemness, foster drug resistance, facilitate metastasis, and aid immune evasion, all of which highlight their significance in cancer progression and treatment challenges. A deeper understanding of these mechanisms could reveal new therapeutic targets.

### Effect of EVs released by cancer-associated fibroblasts on CSCs

Cancer-associated fibroblasts (CAFs) are key components of the TME in solid tumors, displaying significant heterogeneity in their phenotype, origin, and function. CAFs can originate from various sources including native fibroblasts reprogrammed by tumor-derived factors, mesenchymal cells recruited from the bone marrow, or cells derived from adipocyte precursors, endothelial cells, mesothelial cells, or pericytes [74]. As primary producers of the extracellular matrix in the TME, CAFs play critical roles that extend to promoting cancer cell proliferation, angiogenesis, and remodeling of the extracellular matrix (ECM). Furthermore, CAFs orchestrate tumor-promoting inflammation and manipulate the immune microenvironment to foster immune suppression. These functions are executed through the release of factors, exosomes, and metabolites that participate in complex signaling interactions with cancer cells, stromal components, and infiltrating immune cells [75].

For instance, exosomes from CAFs secreting netrin-1 can indirectly induce the secretion of cytokines like IL6 and IL8, which in turn promote cancer stemness. It has been observed that CAFs can directly secrete IL-6, and IL-8 stimulates STAT3/Notch signaling pathways to sustain the stemness of tumor cells [75]. In addition, *miR-146a-5p* exosomes secreted by CAF in urothelial bladder cancer can also enhance CSC characteristics and drug resistance by activating STAT3 and mTOR signaling pathways [76]. CAF-derived exosomes (CAF-exo) can also enhance the characteristics of CSCs through the regulation of the TGF-β signaling pathway [77, 78], and the Wnt/β-catenin pathway [79], leading to cancer progression and increased chemotherapy resistance. Factors within the TME, like hepatocyte growth factor (HGF), which binds to the c-MET tyrosine kinase receptor activating β-catenin-dependent transcription, support CSC phenotypes and contribute to tumor heterogeneity by providing suitable niches for CSCs [80]. Hu and colleagues discovered that *miR-92a-3p* in CAF-secreted exosomes targets FBXW7 and MOAP1 in tumor cells, enhancing β-catenin expression, tumor stemness, and EMT, while inhibiting apoptosis and leading to metastasis and chemoresistance in CRC [81]. Additionally, studies have shown that upregulation of PD-L1 in chemotherapy-treated CAFs leads to increased HGF secretion, stimulating cancer progression, enhancing cell invasion and stem cell properties, and inhibiting apoptosis [82]. Moreover, Li et al. found that CAF-secreted HGF could also promote CSC properties through phosphorylation of STAT3 [83]. Moreover, CAF-derived exosomes can also deliver non-coding RNAs to target HIF-1α to regulate stem cell properties and glycolysis, thereby regulating tumor progression [84, 85].

In summary, CAF-exo serve an essential function in sustaining CSC traits and their resistance to drugs. However, there are not many studies on CAF-exo for the management of tumors, possibly due to the complex origins of CAFs and the challenges in identifying the specific cells they target.

### Effects of exosomes released by mesenchymal stem cells on CSCs

MSCs are versatile cells capable of self-renewal and differentiation into various lineages. Numerous studies have shown that MSCs can migrate to tumor sites and influence tumor matrix formation. Moreover, within the TME, interactions between MSCs and CSCs can promote tumor growth and metastasis through various mechanisms [86]. MSCs, known for their high plasticity, can modify their behavior and role depending on the cytokines produced by nearby immune cells, displaying either tumor-promoting or tumor-inhibiting characteristics depending on the tumor environment [87]. The most typical is bone marrow-derived mesenchymal stem cells (BM-MSCs), which can have an impact on stem cell traits in many tumors, such as acute myeloid leukemia [88], triple-negative breast cancer (TNBC) [89], HCC [90], myeloma [91], CRC [92], and pancreatic cancer [93]. BM-MSC-derived exosomes can modulate CSC traits by carrying RNAs or cytokines that impact downstream pathways. For example, BM-MSC-derived exosomal *miR-142-3p* promotes CSC traits by repressing Numb expression and promoting Notch target gene expression [92]. Additionally, BM-MSCs secrete AlkB homolog 5, an exosomal m6A regulator that increases TNBC stem cell traits by upregulating m6A modification of UBE2C and downregulating p53, thereby fostering TNBC growth and metastasis [89]. MSC-derived exosomes (MSC-exo) can both promote and inhibit malignant behaviors in CSCs. For example, exosomes from BM-MSCs containing *circ0030167* suppress CSC traits in pancreatic cancer by targeting the miR-338-5p/Wif1/Wnt8/β-catenin axis [93]. In HCC, MSC-derived exosomal *lncRNAs C5orf66-AS1* inhibit malignant behaviors like self-renewal, stemness, invasion, and migration by targeting the *miR-127-3p*/DUSP1/ERK axis [90]. Another study also showed that MSC-derived EV, prostaglandin D2 synthase (L-PGDS) reduced the levels of stem cell markers (such as Oct4, Nanog, and Sox2) and suppressed the phosphorylation of STAT3, thereby affecting CSC properties and tumor progression [94]. Thus, MSC-exo not only regulates CSCs but also regulates tumor immunity and drug resistance. MSC-exo has been extensively studied for its roles in regulating tumor immunity, resistance, and stemness, making it a focal point in cancer treatment research due to its significant potential and advantages [95].

### Effects of exosomes released by other cells within the TME on CSCs

In the TME, not only tumor cells, CAFs and MSCs can act on CSCs, but exosomes secreted by other cells like TAMs, myeloid-derived suppressor cells (MDSCs), and adipose-derived stem cells can also regulate CSCs. Among them, M2 macrophages in TAMs primarily facilitate immune evasion, angiogenesis, tissue remodeling, and resistance to chemoradiotherapy, which in turn support tumor proliferation, growth, and metastasis. For example, exosomal *miR-27a-3p* secreted by M2 macrophages boosts liver CSC traits by reducing the expression of thioredoxin-interacting protein (TXNIP) in liver cancer [96]. Chang et al. found that *miR-21-5p* levels in exosomes derived from TAMs were down-regulated in pancreatic cancer, inhibiting Nanog/Oct4 expression, and targeting Krüppel-like factor 3 (KLF3) decreased, thereby inhibiting CSC activity in pancreatic cancer [97].

MDSCs are heterogeneous populations of immature myeloid cells that mainly shape immune responses to support tumor niches, with their mechanisms being complex and encompassing both immunosuppressive and non-immune roles, such as enhancing stemness and promoting angiogenesis [98]. MDSC-derived exosomes can stimulate and maintain CSC properties through IL-6/STAT3 and NO/NOTCH signaling pathways [99, 100]. Furthermore, bone marrow-derived telocytes and their mitochondria have been shown to influence CSC traits via *miR-146a-5p*, which mainly affects the expression of EMT markers (N-cadherin, vimentin, E-cadherin) and tumorigenic markers (BRCA1, P53, SOX2) in CSCs [101]. Numerous studies have demonstrated that *miR-146a-5p* communicates information through exosomes, such as CAF [76] and melanoma cells [102], potentially including communication between telocytes and CSCs in this context.

**Fig. 3:**
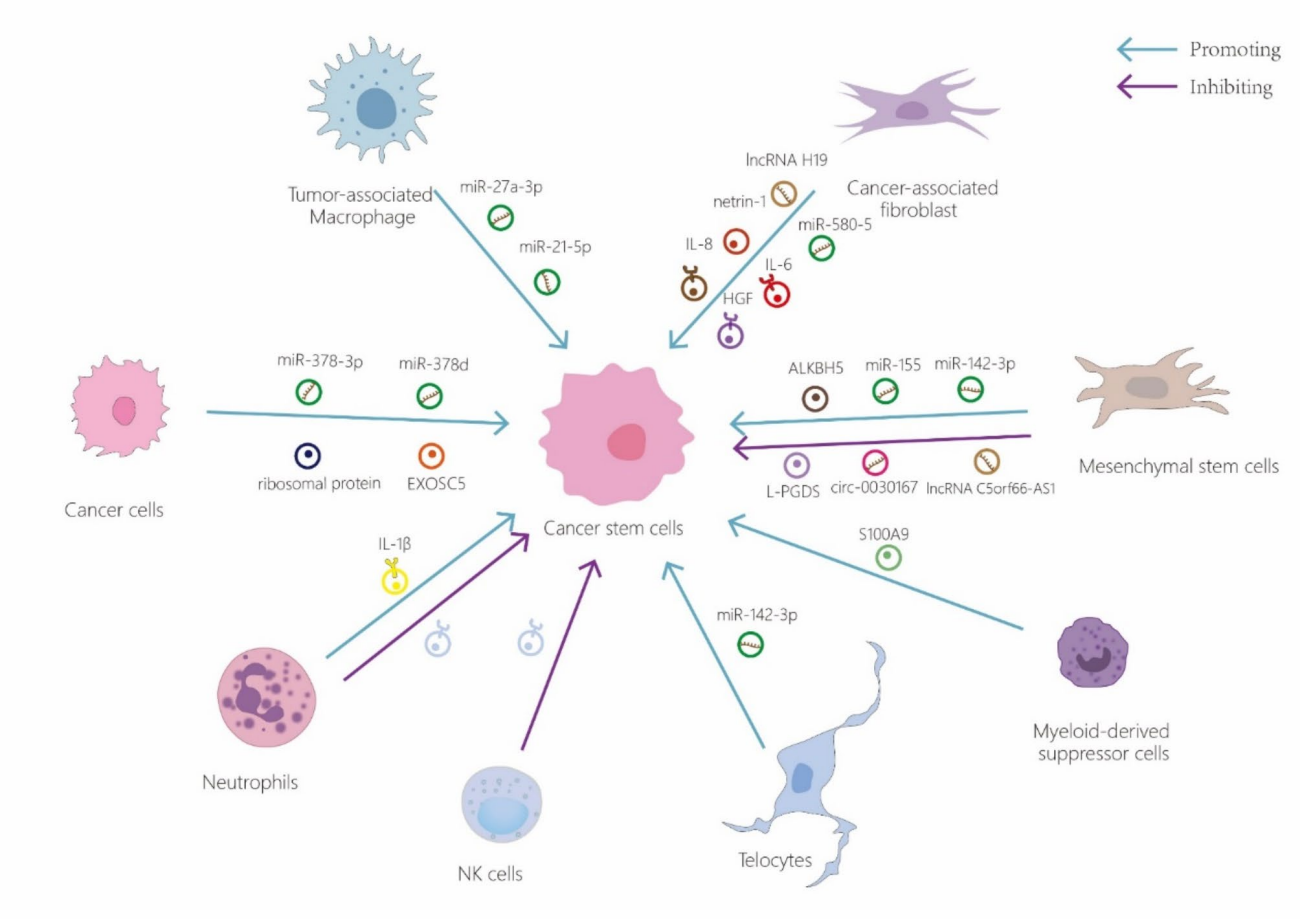
Other cells in the tumor microenvironment also secrete exosomes that impact cancer stem cells

Exosomes secreted by NK cells (NK-exo) are enriched with various bioactive molecules, including cytotoxic proteins like perforin and Fas/FasL, which can stimulate apoptosis in cancer cells. Additionally, the microRNAs contained within these exosomes can suppress gene expression, leading to reduced cell proliferation and enhanced apoptosis in cancer cells [103]. At present, the majority of research on NK-exo focuses on their impact on tumor and immune cells, with limited studies exploring their effects on the relatively small population of CSCs within tumors. Future investigations are expected to further elucidate their interactions. Recent findings have demonstrated that canine NK-exo can inhibit tumor growth by downregulating CSC-associated markers and augmenting the tumor suppressor activity of p53 [104]. Therefore, under existing targeted immunotherapy with NK-exo against tumor cells, NK-exo immunotherapy with more precise targeting of CSC may be just around the corner (Fig. 3) Table 2.

## Significance of CSCs and exosomes for clinical treatment

### Targeting CSC-exo for tumor therapy

The interaction between CSC-exo and other elements in the TME is pivotal for tumor progression, making their integration into precision therapy a crucial clinical objective. By targeting CSCs and disrupting the communication between CSC-exo and the TME, we could potentially impede tumor progression, including the acquisition of stemness, invasiveness, and drug resistance by cancer cells, thus enhancing therapeutic outcomes. Research focused on the direct effects of modulating exosome development is expanding and shows promise as a treatment modality. Studies have indicated that IFN-α can decrease EV secretion in subsets of malignant melanoma CSCs, significantly reducing their tumorigenicity and stemness, as well as downregulating the expression of several oncogenic miRNAs in exosomes, including *miR98-5p*, *miR-191*, *miR-744-3p*, and *let-7e-3p* [105]. Pharmacological interventions targeting specific pathways to reduce CSC-exo secretion are also being explored. For instance, aspirin has been shown to inhibit exosome release under hypoxic conditions via the HIF-1α/COX-2 pathway, consequently reducing CSC traits and tumor proliferation, migration, and angiogenesis [106]. Furthermore, Chen et al. discovered that targeting CSC-EVs with Ovatodiolide treatment sensitized CSCs to cisplatin, primarily by inhibiting oral squamous cell carcinoma tumorigenesis through a reduction of CSC-EV cargo, including mTOR, PI3K, STAT3, β-catenin, and *miR-21-5p* [107]. While numerous studies are directed towards reducing CSC-exo release to mitigate CSC-related harm, currently, there appears to be no research employing CSC-exo as a treatment modality for tumors Table 3.

The biology of exosome biogenesis and release is inherently intricate, involving numerous pathways such as the endosomal sorting complexes required for transport (ESCRT) machinery, tetraspanins, and lipid raft-associated mechanisms. Specifically targeting these pathways in CSCs without impacting normal cells presents significant challenges due to the commonality and overlap of exosome production mechanisms across various cell types. Systemic inhibition of these pathways could unintentionally disrupt normal physiological processes that rely on exosome communication. Additionally, many of the pathways integral to exosome release are also vital for the function of normal stem cells and other healthy tissues. Non-specific suppression of exosome release might lead to off-target effects and toxicity, potentially impairing normal cellular communication, immune functions, and tissue homeostasis. This raises substantial safety concerns, complicating the translation of these strategies into clinical settings. Furthermore, developing treatments that can specifically target exosome release from CSCs without affecting other cells remains a considerable technical hurdle. Current methods lack the precision necessary to selectively inhibit exosome release from CSCs, which is crucial to prevent damage to normal cells. Advances in targeting technologies are needed to achieve the specificity required for effective and safe clinical use. Additionally, CSCs display significant heterogeneity within and between different tumor types. This diversity complicates the task of identifying universal targets for inhibiting exosome release, as different CSC subpopulations may depend on distinct exosomal pathways. Personalized approaches would be essential, necessitating comprehensive characterization of CSCs in each patient, which is resource-intensive and currently not feasible for broad clinical application. Present therapeutic strategies that focus on CSCs typically aim at direct targeting and elimination of these cells through cytotoxic agents, immunotherapy, or differentiation therapy. These approaches have demonstrated more immediate and observable benefits in preclinical and clinical trials. Therefore, the development and refinement of these strategies are prioritized over the emerging field of targeting CSC-exo release. Hence, overcoming these challenges will require significant advancements in our understanding of exosome biology, improved targeting techniques, and thorough evaluation of the efficacy and safety of these methods to make the modulation of CSC-exo release viable for widespread clinical application.

### Targeting exosomes secreted by other cells in the TME for tumor therapy

In tumor therapy, targeting cell-secreted exosomes within the TME presents a promising avenue, particularly the application of MSC-exo in combating CSCs. MSC-exo possess unique properties that allow them to effectively target CSCs, potentially enhancing cancer treatment outcomes. These exosomes can be engineered to carry therapeutic agents directly to CSCs, including chemotherapeutic drugs, small interfering RNAs (siRNAs), or miRNAs that target critical pathways for CSC survival and proliferation. This targeted strategy helps minimize the systemic toxicity typically associated with traditional chemotherapy, thus reducing adverse side effects. For instance, exosomes derived from *GRP7*-siRNA modified BMSCs can enhance the responsiveness of HCC cells to sorafenib. The combination of exosomes from *GRP78*-siRNA modified MSCs and sorafenib effectively inhibits the proliferation and invasion of HCC cells in vitro [108]. MSC-exo loaded with miRNAs that downregulate drug resistance genes can restore the sensitivity of CSCs to existing treatments, improving their efficacy. For example, MSC-derived exosomes overexpressing *miR-199a* can suppress glioma growth, invasion, and migration while boosting sensitivity to temozolomide both in vitro and in vivo [109]. Another example involves MSC-exo recombinant *miR-193a*, which can inhibit tumor colony formation, invasion, migration, and proliferation, and induce apoptosis in resistant cells by downregulating LRRC1 [110]. Additionally, modifying MSCs can change the signaling properties of their exosomes, reducing their pro-tumorigenic signals. For example, reducing TGF-β1 expression in MSCs can diminish their exosomes’ ability to mediate EMT while enhancing their capability to inhibit cell proliferation and promote apoptosis [111]. Interestingly, past studies have shown that MSC-exo can actively modulate immune responses within the TME. These exosomes contain immunomodulatory molecules that bolster anti-tumor immunity, which can be leveraged to modulate the tumor immune microenvironment for more effective immunotherapy. For instance, BMSC-exo loaded with galectin-9 siRNA and the oxaliplatin (OXA) prodrug can modulate macrophage polarization, attract cytotoxic T lymphocytes, and decrease Treg levels, thereby triggering a robust anti-tumor immune response and improving immunotherapy outcomes in vitro and in vivo [112].

Therapeutic approaches utilizing CAF-derived exosomes predominantly aim to convert CAFs from promoting tumors to inhibiting them. This transformation can be achieved through drugs, cytokines, or genetic modifications that modify CAF behavior. For instance, in pancreatic ductal adenocarcinoma, using the vitamin D receptor agonist calcipotriol to reprogram stromal cells has shown to curtail tumor growth and improve the efficacy of pancreatic cancer treatments [113]. Such reprogrammed CAFs might release exosomes that possess anti-tumor characteristics. CAFs have the capability to directly influence immune cell activity and indirectly lead to immune effector cell dysfunction by elevating immune checkpoint molecules on their surfaces and restructuring the ECM within the TME. Additionally, CAFs can suppress immune cell-driven anti-tumor responses through the release of various chemokines, cytokines, and other effector molecules [114]. As with other exosomes, modifying the contents of CAF-derived exosomes to include anti-tumor molecules is another promising approach.

Exosomes from DCs, T cells, NK cells, and other immune cells are packed with proteins, lipids, and nucleic acids that can activate and modulate the immune system. These immune cells can target CSCs or tumor cells directly and indirectly affect them by altering the immune microenvironment, thereby playing a role in tumor therapy. Exosomes from DCs are particularly effective as they are powerful antigen-presenting vesicles that can induce robust anti-tumor immune responses. They transport tumor antigens, MHC molecules, and co-stimulatory signals, efficiently priming T cells to initiate strong cytotoxic attacks on tumor cells. Clinical trials have shown that vaccines based on DC-derived exosomes can trigger tumor-specific immune responses, marking an innovative and promising direction in cancer immunotherapy. These exosomes’ ability to present antigens and activate T cells positions them as excellent candidates for creating personalized cancer vaccines [115]. Li et al. developed a nanovaccine platform utilizing DC-derived exosomes loaded with neoantigens, which considerably curbed tumor growth, extended survival, delayed tumor onset, and fostered long-term memory, thus preventing tumor recurrence and metastasis while enhancing survival rates [116].

T cell-derived exosomes (T-exo) contain proteins and RNA, reflecting the activation state and cytotoxic potential of T cells. These exosomes can transfer functional molecules such as granzyme, perforin, and cytokines to target cells, inducing tumor cell apoptosis. Additionally, they can regulate the TME by promoting immune cell infiltration and enhancing the antitumor activity of other immune cells. Leveraging T-exo can amplify the antitumor immune response, thereby increasing the efficacy of T cell therapies and checkpoint inhibitors [117] Zhu et al. developed hybrid nanovesicles by merging exosomes from bispecific CAR-T cells, which target mesothelin and PD-L1, with liposomes to administer paclitaxel (PTX) for treating metastatic lung cancer. These nanovesicles blocked PD-L1 on tumors, reducing T cell exhaustion and enhancing the anticancer effects by facilitating PTX-induced immunogenic cell death [118].

The potential of NK-exo in cancer therapy is notable due to their capacity to specifically target and eradicate cancer cells, including CSCs. NK-exo possess inherent cytotoxic properties attributed to the inclusion of perforin and granzyme B, which can trigger apoptosis in cancer cells. This capability enables them to target CSCs, which are frequently resistant to standard treatments, thus overcoming a significant hurdle in cancer therapy. Additionally, NK-exo can boost the effectiveness of existing treatments by making tumor cells more susceptible to chemotherapy and radiotherapy. They also play a role in altering the TME, creating conditions that are unfavorable for CSCs and cancer cells. Moreover, NK-exo can activate other immune cells such as T cells and macrophages by transferring proteins and RNA, which helps to initiate an immune response. This immunomodulatory function can amplify the overall anti-tumor response and complement other immunotherapeutic strategies. The typically immunosuppressive TME can impair the success of immunotherapies, but remodeling the TME with NK-exo can significantly improve immunotherapy outcomes. NK-exo can also be integrated with other therapeutic approaches to enhance their impact, for example, they can be combined with checkpoint inhibitors to counteract tumor immune evasion tactics. Their inherent ability to target and destroy cancer cells positions them as promising agents for developing exosome-based immunotherapies. Additionally, these exosomes can be engineered to transport chemotherapeutic drugs, RNA interference molecules, or CRISPR/Cas9 components, targeting specific genetic mutations in cancer cells. This multifaceted approach could lead to more thorough and effective cancer treatment strategies [119].

A therapeutic strategy involving TAM-derived exosomes in cancer treatment focuses on converting TAMs from a tumor-promoting M2 phenotype to an anti-tumor M1 phenotype. This transformation can be accomplished using drugs, cytokines, or genetic interventions that influence macrophage polarization. For example, Chen et al. developed engineered exosomes containing siPDL, specifically targeting PDL1 on M2 TAMs, which reprogrammed them into anti-tumor M1 macrophages and reversed the immunosuppressive TME [120]. Exosomes from these reprogrammed M1 macrophages convey anti-tumor signals within the TME and to CSCs, thereby inhibiting tumor growth and improving the effectiveness of existing cancer treatments. Additionally, Kim et al. utilized exosomes derived from M1 macrophages to trigger a phenotypic switch in M2-like TAMs, shifting them from an anti-inflammatory to a pro-inflammatory M1 macrophage state. These reprogrammed M1 macrophages exhibited protein expression profiles characteristic of classical M1 macrophages, enhanced their phagocytic function and cross-presentation capabilities, and significantly bolstered anti-tumor immunity near the tumor site [121].

In certain instances, exosomes released by neutrophils (N-exo) can promote tumor growth by facilitating angiogenesis and dampening immune responses. Therapeutic approaches could involve blocking the release or uptake of these pro-tumorigenic exosomes. By targeting pathways critical to N-exo mediated communication, it may be feasible to interrupt the supportive interactions between neutrophils and tumor cells, thus impeding tumor progression. Additionally, N-exo can be utilized to bolster anti-tumor immune responses. By transmitting pro-inflammatory signals and activating other immune cells, these exosomes can contribute to creating a more inhospitable environment for tumor cells. This strategy could complement other immunotherapies, such as checkpoint inhibitors, to amplify the overall immune response against tumors. Another therapeutic avenue involves using N-exo to directly target CSCs by incorporating miRNAs or other molecules that suppress CSC characteristics into N-exo. This method targets the fundamental factors behind tumor recurrence and metastasis, aiming to enhance long-term cancer treatment outcomes. For instance, Zhang et al. harvested N-exo with enhanced tumor-targeting capabilities and combined them with DOX to significantly enhancing the efficacy of tumor treatment [122].

In conclusion, the rationale behind utilizing exosomes to target non-CSCs and non-CSCs-derived exosomes for tumor therapy lies in the complex and dynamic nature of TME, where interactions between CSCs and non-CSCs play a vital role in sustaining the CSC niche and enhancing tumor diversity. Non-CSCs, while not initiating tumors themselves, support CSC activities through mechanisms like paracrine signaling, extracellular matrix modification, and immunomodulation. Disrupting these supportive interactions via exosome-mediated delivery of therapeutic agents or siRNAs that target specific signaling pathways in non-CSCs can destabilize the CSC niche, potentially leading to CSC depletion or re-differentiation. By focusing on non-CSCs, which make up the majority of the tumor, exosome-based treatments could decrease the overall tumor mass and indirectly hinder CSC functionality. This approach may result in more sustained and effective cancer treatment outcomes, possibly preventing tumor recurrence and metastasis, often driven by CSC persistence. Moreover, this method could be integrated with other CSC-specific therapies to improve therapeutic efficacy. Ultimately, targeting non-CSCs with exosomes presents a novel and supplementary approach in CSC-centric cancer therapy, aiming to undermine the supportive role of non-CSCs within the TME, reduce tumor diversity, and enhance the effectiveness of cancer treatments Table 4.

### Targeting CSCs with exosomal drug delivery

As our research on exosomes and EVs has become more and more intensive, exosomes have now emerged as carriers and engineered EVs to explore cancer therapy. Exosomes offer numerous advantages over traditional delivery systems like liposomes, viral vectors, and polymers, including high biocompatibility, stability, specific homing capabilities, low toxicity, and the ability to cross the blood-brain barrier [123]. These properties enable more precise drug delivery to target cells and substantially boost the effectiveness of cancer treatment. At present, more and more studies on engineered EVs are becoming more and more mature, and many studies have explored engineered EVs to target CSC for cancer therapy. For example, some researchers have engineered EVs to perform a dual knockdown of IQGAP1 and FOXM1, coupled with sorafenib treatment. This combination has shown to disrupt key signaling pathways such as PI3K/Akt and MAPK/ERK, improving sorafenib’s efficacy in tumor treatment by preventing β-catenin’s nuclear translocation, essential for CSC maintenance. These engineered EVs demonstrate enhanced targeting and homing capabilities compared to sorafenib alone, reducing CSC populations and reversing tumor resistance, thereby enhancing the efficacy of cancer therapy [124]. Moreover, Ishiguro et al. have developed engineered EVs targeting liver CSCs expressing EpCAM, facilitating the delivery of siRNA to β-catenin. This approach depletes β-catenin expression, curtails CSC proliferation, and achieves therapeutic effects. EpCAM is not only a CSC marker in the liver and other epithelial tissues but also a transcriptional target of the canonical Wnt/β-catenin signaling pathway that regulates liver CSC proliferation [125]. In recent years, nanomaterials with tumor therapeutic potential have emerged as effective therapeutic tools, with exosomes providing more precise tumor targeting than other carriers. Yong and colleagues have developed biocompatible tumor-cell-exocytosed exosome-biomimetic porous silicon nanoparticles (PSiNPs) for encapsulating doxorubicin (DOX). These PSiNPs, first loaded with DOX through endocytosis by tumor cells (termed DOX@-PSiNPs), are then exocytosed, resulting in exosome-sheathed doxorubicin-loaded PSiNPs (DOX@E-PSiNPs). Treatment with DOX@E-PSiNPs shows superior intracellular abundance and more effectively inhibits CSC spheroid number and size compared to other forms. Moreover, DOX@E-PSiNPs exhibit strong cytotoxicity targeted specifically at tumor cells and CSCs, with minimal toxicity to normal cells, and do not trigger immune responses in various mouse models, further confirming the high biocompatibility and safety of exosomes [126].

Targeted drug delivery to CSCs via exosomes marks a groundbreaking advancement in contemporary cancer treatment, tackling many limitations of conventional therapies. This method holds considerable clinical promise, notably in boosting the effectiveness of cancer therapies while lessening side effects, thereby improving patient outcomes. A primary benefit of this exosome-mediated approach is its ability to circumvent therapeutic resistance. CSCs notoriously resist standard chemotherapies and radiation due to their dormant nature, proficient DNA repair capabilities, and drug efflux transporter expression. These traits allow CSCs to withstand usual treatments, leading to tumor resurgence and spread. Exosome-based delivery systems, however, can be tailored to encapsulate potent anticancer drugs and specifically target CSCs, directly tackling the fundamental causes of treatment resistance. Successfully eliminating CSCs greatly diminishes the likelihood of tumor recurrence and metastasis, promoting more enduring and long-term therapeutic results. Additionally, exosome-mediated drug delivery offers enhanced precision and specificity compared to conventional treatment methods. By bioengineering, exosomes can be customized to recognize and bind to specific CSC markers like CD133, CD44, and ALDH. This targeting ability ensures that drugs are delivered directly to CSCs within a diverse tumor environment, minimizing unintended impacts and reducing toxicity to normal stem cells and surrounding healthy tissues. This targeted approach is particularly vital for decreasing the harmful side effects commonly associated with traditional chemotherapy, which indiscriminately impacts both cancerous and non-cancerous cells. The biocompatibility and low immunogenicity of exosomes also make them ideal candidates for clinical applications. Unlike synthetic drug delivery systems, exosomes are natural vesicles derived from cells, reducing the risk of eliciting immune responses. This characteristic enhances their safety profile, making them suitable for repeated administration, which may be necessary for effectively eradicating CSCs. Using patient-derived exosomes further minimizes the risk of adverse immune reactions, promoting a personalized medicine approach in cancer treatment. The potential for combination therapies is another crucial feature. Exosomes can carry a mix of therapeutic agents, including chemotherapeutics, RNA-based treatments, and proteins, facilitating a comprehensive assault on CSCs. This multi-drug strategy can enhance therapeutic efficacy synergistically, as different agents target multiple survival pathways in CSCs, countering these cells’ adaptability and redundancy. Moreover, exosomes can be engineered to alter the TME, making it less supportive of CSC survival and proliferation. The clinical translation of exosome-mediated targeted drug delivery systems to CSCs also represents a step forward in personalized medicine. By isolating and engineering exosomes from a patient’s own cells, treatment regimens can be tailored to the specific biological characteristics of the patient’s tumor, enhancing therapeutic effectiveness and minimizing adverse effects. This personalized approach not only betters patient outcomes but also shifts the paradigm in the development and application of cancer therapies.

In conclusion, utilizing exosomes as drug delivery vehicles targeting CSCs holds significant potential for revolutionizing cancer treatment. These exosome-mediated systems tackle challenges like therapeutic resistance, improve targeting accuracy, guarantee biocompatibility, facilitate combination therapies, and accommodate personalized treatment approaches. This innovative method offers a promising avenue for effectively combating cancer and enhancing patient outcomes.

## Conclusion and prospects

CSCs are pivotal in driving tumor progression and are often resistant to standard treatments, which can lead to tumor recurrence and metastasis. Recent advances in the study of CSCs and exosomes highlight the critical role of CSC-exo in tumor dynamics. Consequently, there is a focused effort to counter the detrimental impacts of CSCs by targeting CSC-exo. Moreover, the natural carrier capabilities of exosomes are being exploited to deliver therapeutic agents specifically to CSCs, aiming for precise and effective treatment strategies that could potentially eliminate CSCs.

Despite these promising theoretical approaches, several challenges persist in the research and practical application of strategies targeting CSCs and CSC-exo. Current methods for identifying and isolating CSCs rely primarily on surface markers, which may lack precision. Moreover, standardization is needed in the extraction methods for CSC exosomes to reduce the potential interference from exosomes of other origins. Additionally, as CSCs and normal stem cells share certain signaling pathways, developing therapeutic strategies that specifically target CSCs without affecting normal stem cells remains a significant scientific challenge.

Non-CSCs, while not directly initiating tumors, are crucial in maintaining the CSC niche and facilitating cancer progression. Hence, utilizing exosomes to target non-CSCs could indirectly affect CSC dynamics, potentially disrupting the supportive TME and diminishing tumor recurrence. By inhibiting critical signaling pathways used by non-CSCs to support CSCs, the CSC niche may become destabilized, which could decrease CSC survival, proliferation, and differentiation capabilities. This might enhance the effectiveness of therapies aimed at CSCs, which typically face challenges due to the intrinsic adaptability and resilience of CSCs. However, significant hurdles are associated with this approach, particularly concerning the role of exosomes secreted by non-CSCs. These exosomes can transport various bioactive molecules that might unintentionally promote CSC survival, stemness, and metastatic capabilities. For instance, non-CSC-derived exosomes might carry growth factors, cytokines, or pro-inflammatory signals that sustain the CSC phenotype or protect CSCs from apoptosis. Hence, the dual function of exosomes from non-CSCs as both potential therapeutic vectors and contributors to CSC resilience adds complexity to this therapeutic strategy. The risk of exosome-based therapies inadvertently supporting CSC survival highlights the need for careful design and rigorous testing of these approaches. Potential strategies to address these challenges may involve engineering exosomes with highly specific targeting ligands, developing combination treatments that target both CSCs and non-CSCs, and utilizing advanced drug delivery systems to control the release and localization of therapeutic agents. Ultimately, achieving precise targeting, leveraging exosomes effectively, and accurately focusing treatments on CSCs are believed to be promising directions for improving clinical cancer treatment.

**Table 1:** Examples of exosomes derived from cancer stem cells discussed in this review

Exosomal cargo	Source of exosomes	Target cells	Effect of tumor progression	Mechanisms affecting tumors	Reference
Notch1 protein	Glioblastoma CSC	Glioma cell	Promoting tumor stemness	Activating the Notch1 signaling	[14]
*lncRNA FMR1-AS1*	Esophageal CSC	esophageal cancer cell	Promoting tumor stemness	Activating TLR7-NFκB pathway	[16]
*miR-146a-5p*	Colorectal CSC	Colorectal cancer cell	Promoting tumor stemness	Targeting Numb to regulate the Wnt-β-catenin pathway	[17]
*lncRNA DOCK9-AS2*	Thyroid CSC	Thyroid carcinoma cells	Promoting tumor stemness	Activating Wnt/β-catenin pathway	[18]
*circ-ZEB1*	Liver CSC	Liver cancer cell	Promoting tumor stemness and metastasis	Increasing the expression of stemness marker CD133 and downregulating E-cadherin and EpCAM	[19]
*circ-AFAP1*	Liver CSC	Liver cancer cell	Promoting tumor stemness and metastasis	Increasing the expression of stemness marker CD133 and downregulating E-cadherin and EpCAM	[19]
*miR-26a*	Glioblastoma CSC	Microvessel endothelial cell	Promoting tumor angiogenesis	Targeting PTEN and activating the PI3K/Akt signaling pathway	[23]
*miR-21*	Glioblastoma CSC	Endothelial cell	Promoting tumor angiogenesis	Upregulating VEGF expression	[24]
VEGF-A	Glioblastoma CSC	Endothelial cell	Promoting tumor angiogenesis	Promoting angiogenesis of endothelial cells	[25]
*lncRNA MALAT1*	Thyroid CSC	Thyroid cell	Promoting tumor invasion	Inducing EMT	[34]
linc-ROR	Thyroid CSC	Thyroid cell	Promoting tumor invasion	Inducing EMT	[34]
SLUG	Thyroid CSC	Thyroid cell	Promoting tumor invasion	Inducing EMT	[34]
*lncRNA CDKN2B-AS1*	Thyroid CSC	Thyroid cancer cell	Affected tumor growth and metastasis	Decreasing the expression of E-cadherin and increasing the expression of P4HA1	[36]
*miR-210-3p*	Lung CSC	Lung cancer cell	Promoting migration and invasion	Upregulating the expression of N-cadherin, vimentin, MMP-9, and MMP-1 in lung cancer cells, and downregulating E-cadherin expression	[37]
*miR-197*	Breast CSC	Breast cancer cell	Promoting tumor growth and metastasis	Facilitating EMT and inhibiting PPARG expression	[127]
*miR-19b-3p*	CSC of clear cell renal cell carcinoma	Clear cell renal cell carcinoma cell	Promoting tumor proliferation and metastasis	Inducing EMT via repressing the expression of PTEN.	[38]
*miR-4535*	Melanoma CSC	Melanoma cell	Promoting tumor metastasis	Inhibiting autophagy pathway	[39]
*miR-1268a*	Melanoma CSC	Melanoma cell	Promoting tumor metastasis	Inhibiting autophagy pathway	[40]
*lncRNA Mir100hg*	Lung CSC	Lung cancer cell	Promoting tumor metastasis	Targeting *miR-15a-5p* and *miR-31-5p* and increasing the glycolytic activity	[43]
*lncRNA UCA1*	Oral CSC	Oral squamous cell carcinoma and Macrophage	Facilitating tumor progression and immunosuppression	Promoting M2 macrophage polarization and Suppressing CD4^+^T cell activity by Targeting LAMC2^+^	[54]
O-GlcNAc transferase	Esophageal CSC	CD8^+^T cell	Promoting cancer immunosuppression	Protecting esophageal carcinoma stem cells from CD8^+^T cells through up-regulation of PD-1	[56]
*miRNA-146a-5p*	Colorectal CSC	Colorectal cancer cell	Promoting tumor stemness and immunosuppression	Targeting Numb	[57]
tenascin-C	Brain CSC	T cell	Promoting tumor immunosuppression	Targeting the integrin receptors α5β1 and αvβ6 and attenuating T cell mTOR pathway signaling	[58]
*miR-210*	Pancreatic CSC	Pancreatic cancer cell	Promoting tumor drug resistance	Activating mTOR signaling pathway	[61]
*miR-155*	Breast CSC	Breast cancer cell	Promoting tumor drug resistance	Downregulating of C/EBP-β and inhibiting the expression of TGF-β, C/EBP-β, and FOXO3a	[62]
*MALAT1*	Glioma CSC	Microglia	Promoting tumor immunosuppression, resistance, growth, and angiogenesis	Regulating the *miR-129-5p*/HMGB1 axis and modulating LPS and polarizing microglia to an M2 phenotype and influencing the secretion of IL-6, IL-8, and TNF-α.	[63]
ANXA6	Breast CSC	Breast cancer cell	Promoting tumor drug resistance	Regulating the YAP1/Hippo pathway	[64]

**Table 2:** Examples of exosomes from different sources within the tumor microenvironment that target cancer stem cells discussed in this review

Exosomal cargo	Source of exosomes	Mechanisms affecting tumors	Effect of tumor progression	Reference
*miR-378a-3p /miR-378d*	Breast cancer cell	Targeting of DKK3 and NUMB and activating the WNT and NOTCH stem cell pathways	Promoting tumor drug resistance	[71]
EXOSC5	Endometrial carcinoma cell	Increasing NTN4 expression and activating c-MYC via the integrin β1/FAK/SRC pathway	Promoting tumor stemness	[72]
ribosomal protein	Non-stem bladder cancer cell	Promoting the expression of stemness factors	Promoting tumor stemness	[73]
netrin-1	Colon and lung CAF	trigger the secretion of IL6	Promoting tumor stemness	[75]
*miR-146a-5p*	Cancer-associated fibroblast of urothelial bladder cancer	Activating STAT3 and mTOR signaling pathways	Promoting tumor stemness and drug resistance	[76]
*lncRNA H19*	CAF of colorectal cancer	Activating the β-catenin pathway	Promoting tumor stemness and drug resistance	[79]
*miR-92a-3p*	CAF of colorectal cancer	Targeting FBXW7 and MOAP1, and promoting the β-catenin expression	Promoting tumor metastasis and chemoresistance	[81]
*miR-7641*	CAF of breast cancer	Targeting the HIF-1α pathway suppressing glycolysis	Inhibiting tumor stemness	[84]
*circHIF1A*	CAF of breast cancer	Sponging *miR-580-5p* and targeting CD44 expression	Promoting tumor stemness	[85]
*miR-142-3p*	BM-MSC /stromal cell	Promoting the Notch signaling pathway by downregulating Numb.	Promoting tumor stemness	[92]
ALKBH5	BM-MSC in triple-negative breast cancer	Upregulating UBE2C and downregulating p53	Promoting tumor stemness, growth, and metastasis	[89]
*circ_0030167*	BM-MSC in pancreatic cancer	Sponging *miR-338-5p* and targeting the Wif1/Wnt8/β-catenin axis	Inhibiting tumor invasion, migration, proliferation, and stemness	[93]
*lncRNAs C5orf66-AS1*	MSC in hepatocellular carcinoma	Targeting *C5orf66-AS1/miR-127-3p*/DUSP1/ERK axis	Inhibiting tumor proliferation, migration, invasion, angiogenesis-stimulating	[90]
L-PGDS	MSC in gastric cancer	reducing the levels of stem cell markers and suppressing the phosphorylation of STAT3	Inhibiting tumor stemness	[94]
*miR-27a-3p*	M2 macrophage in hepatocellular carcinoma	downregulating TXNIP	Promoting tumor stemness, proliferation, drug resistance, migration, invasion	[96]
*miR-21-5p*	M2 macrophage in pancreatic cancer	Mediating KLF3	Promoting tumor stemness	[97]
S100A9	Myeloid-derived suppressor cell in colorectal cancer	Enhancing the phosphorylation of STAT3 and NF-κB	Promoting tumor stemness	[99]

**Table 3:** Therapeutic modalities affecting exosomes secreted by the cancer stem cells discussed in this review

Substances related to exosomes	Targeting cell	Effects on exosomes	Effects on tumor progression	Function model	Reference
IFN-α	Malignant melanoma CSC	Affecting exosome production and cargo	Reducing CSC formation and stemness properties	Down-regulating the expression of *miR98-5p*, *miR-191*, *miR-744-3p*, and *let-7e-3p*	[105]
Aspirin	non-small cell lung cancer cell or CSC	Affecting exosome production and cargo	Suppressing of stemness through inhibiting of exosome secretion by CSC	Targeting HIF-1α/COX-2 pathway	[106]
Ovatodiolide	OSCC CSC	Affecting exosome cargo	Suppressing CSC self-renewal, reducing drug resistance	Inhibiting the expressions of *miR-21-5p*, STAT3, and mTOR in CSC-exo.	[107]

**Table 4:** Therapeutic modalities using exosomes discussed in this review

Therapeutic exosome	Parental cell	Function material	Targeting cell	Effect	Reference
*GRP7*-siRNA load exosome	Mesenchymal stem cell	*GRP7*-siRNA	HCC cell	Inhibiting the growth and invasion of the cancer cells in vitro and inhibiting the growth and metastasis of the cancer cells in vivo.	[108]
Exosomes overexpressing miR-199a	Mesenchymal stem cell	*miR-199a*	Glioma cell	Suppressing the proliferation, invasion and migration of glioma cell	[109]
miR-193a load exosome	Mesenchymal stem cell	*miR-193a*	Non-small cell lung cancer cell	Suppressing the colony formation, invasion, migration, and proliferation and promoting apoptosis of resistant cell	[110]
Electroporation-loaded galectin-9 siRNA with oxaliplatin	Mesenchymal stem cell	galectin-9 siRNA, oxaliplatin	Macrophage and cytotoxic T lymphocyte	Eliciting anti-tumor immunity	[112]
Neoantigens load exosome	Dendritic cell	Neoantigens	Melanoma cell	Inhibiting tumor growth, prolonging survival, delaying tumor onset, inducing long-term memory, and preventing recurrence and metastasis	[116]
Chimeric antigen receptor-T (CAR-T) cell-derived exosomes with paclitaxel	T cell	Mesothelin and PD-L1	Lung cancer cell	Promoting immunogenic cell death	[118]
Exosomes erived from M1-type macrophage	M1-type macrophage	-	M2-type macrophage	Leading to the transformation of human patient-derived macrophage into M1-like macrophages	[121]
Doxorubicin load exosome	Neutrophil	Doxorubicin	Gastric cancer cell	Restraining tumor growth	[122]

## Data Availability

Not applicable.

## Abbreviations

ANXA6: Annexin-a6
BC: Breast cancer
BCRP: Breast cancer resistance protein
BM-MSCs: Marrow-derived mesenchymal stem cells
CAF-exo: Cancer-associated fibroblast-derived exosome
CAFs: Cancer-associated fibroblasts
CCRCC: Clear cell renal cell carcinoma
C/EBP-β: Ccaat enhancer binding protein β
CRC: Colorectal cancer
CSC-exo: Exosomes secreted by cancer stem cells
CSCs: Cancer stem cells
DCs: Dendritic cells
ECM: Extracellular matrix
ECs: Endothelial cells
EMT: Epithelial-mesenchymal transition
EMT-TFs: Epithelial-mesenchymal transition-inducing transcription factors
EpCAM: Epithelial cell adhesion molecule
ESCRT: endosomal sorting complexes required for transport
ER: Endoplasmic reticulum
ESEs: Early sorting endosomes
EVs: Extracellular vesicles
FOXO3a: Forkhead box o3
GSCs: Glioblastoma stem cells
HCC: Hepatocellular carcinoma
HGF: Hepatocyte growth factor
HMGB: High mobility group box 1 protein
IFN-γ: Interferon gamma
ILVs: Intraluminal vesicles
KLF3: Targeting krüppel-like factor 3
LPS: Lipopolysaccharide
MDR1: Multidrug resistance-1
MDSC: Myeloid-derived suppressor cells
MHC: Major histocompatibility complex
MMP1: Matrix metalloproteinase 1
MMP9: Matrix metalloproteinase 9
MPO: Myeloperoxidase
MSCs: Mesenchymal stem cells
MVBs: Multivesicular bodies
ncRNA: Non-coding RNA
NK cells: Natural killer cells
OGT: O-glcnac transferase
OPN: Osteopontin
PD-1: Programmed cell death protein 1
PD-L1: Programmed death-ligand 1
P4HA1: Prolyl 4-hydroxylase, alpha polypeptide 1
PTEN: Phosphatase and tensin homologs
PTX: Paclitaxel
TAMs: Tumor-associated macrophages
TGF-β: Transforming growth factor β
TGN: Trans-golgi network
TLR7: Toll-like receptor 7
TME: Tumor microenvironment
TNBC: Triple-negative breast cancer
TNC: Tenascin-c
TNF-α: Tumor necrosis factor-α
TXNIP: Inhibiting thioredoxin-interacting protein
YAP1: Yes-associated protein 1
YB-1: Y-box binding protein 1

## References

[1] Bray F, Laversanne M, Weiderpass E, Soerjomataram I. The ever-increasing importance of cancer as a leading cause of premature death worldwide. Cancer. 2021;127(16):3029–30. 34086348 10.1002/cncr.33587

[2] Bray F, Laversanne M, Sung H, Ferlay J, Siegel RL, Soerjomataram I, et al. Global cancer statistics 2022: GLOBOCAN estimates of incidence and mortality worldwide for 36 cancers in 185 countries. CA Cancer J Clin. 2024;74(3):229–63. 38572751 10.3322/caac.21834

[3] Huang T, Song X, Xu D, Tiek D, Goenka A, Wu B, et al. Stem cell programs in cancer initiation, progression, and therapy resistance. Theranostics. 2020;10(19):8721–43. 32754274 10.7150/thno.41648 PMC7392012

[4] Prasetyanti PR, Medema JP. Intra-tumor heterogeneity from a cancer stem cell perspective. Mol Cancer. 2017;16(1):41. 28209166 10.1186/s12943-017-0600-4 PMC5314464

[5] Lapidot T, Sirard C, Vormoor J, Murdoch B, Hoang T, Caceres-Cortes J, et al. A cell initiating human acute myeloid leukaemia after transplantation into SCID mice. Nature. 1994;367(6464):645–8. 7509044 10.1038/367645a0

[6] Bhat GR, Sethi I, Sadida HQ, Rah B, Mir R, Algehainy N, et al. Cancer cell plasticity: from cellular, molecular, and genetic mechanisms to tumor heterogeneity and drug resistance. Cancer Metastasis Rev. 2024;43(1):197–228. 38329598 10.1007/s10555-024-10172-z PMC11016008

[7] Saw PE, Liu Q, Wong PP, Song E. Cancer stem cell mimicry for immune evasion and therapeutic resistance. Cell Stem Cell. 2024;31(8):1101–12. 38925125 10.1016/j.stem.2024.06.003

[8] Easwaran H, Tsai HC, Baylin SB. Cancer epigenetics: tumor heterogeneity, plasticity of stem-like states, and drug resistance. Mol Cell. 2014;54(5):716–27. 24905005 10.1016/j.molcel.2014.05.015 PMC4103691

[9] Nowell PC. The clonal evolution of tumor cell populations. Science. 1976;194(4260):23–8. 959840 10.1126/science.959840

[10] Peng C, Xu Y, Wu J, Wu D, Zhou L, Xia X. TME-Related biomimetic strategies against Cancer. Int J Nanomed. 2024;19:109–35. 10.2147/IJN.S441135 PMC10773252 38192633

[11] Bejarano L, Jordāo MJC, Joyce JA. Therapeutic targeting of the Tumor Microenvironment. Cancer Discov. 2021;11(4):933–59. 33811125 10.1158/2159-8290.CD-20-1808

[12] Cocozza F, Grisard E, Martin-Jaular L, Mathieu M, Théry C, SnapShot. Extracell Vesicles Cell. 2020;182(1):262–e1. 10.1016/j.cell.2020.04.054 32649878

[13] He G, Peng X, Wei S, Yang S, Li X, Huang M, et al. Exosomes in the hypoxic TME: from release, uptake and biofunctions to clinical applications. Mol Cancer. 2022;21(1):19. 35039054 10.1186/s12943-021-01440-5 PMC8762953

[14] Sun Z, Wang L, Zhou Y, Dong L, Ma W, Lv L, et al. Glioblastoma stem cell-derived Exosomes Enhance Stemness and Tumorigenicity of Glioma cells by transferring Notch1 protein. Cell Mol Neurobiol. 2020;40(5):767–84. 31853695 10.1007/s10571-019-00771-8 PMC11448788

[15] Huang H, Hou J, Liu K, Liu Q, Shen L, Liu B, et al. RAB27A-dependent release of exosomes by liver cancer stem cells induces nanog expression in their differentiated progenies and confers regorafenib resistance. J Gastroenterol Hepatol. 2021;36(12):3429–37. 34258777 10.1111/jgh.15619

[16] Li W, Zhang L, Guo B, Deng J, Wu S, Li F, et al. Exosomal FMR1-AS1 facilitates maintaining cancer stem-like cell dynamic equilibrium via TLR7/NFκB/c-Myc signaling in female esophageal carcinoma. Mol Cancer. 2019;18(1):22. 30736860 10.1186/s12943-019-0949-7 PMC6367809

[17] Hwang WL, Yang MH. Numb is involved in the non-random segregation of subcellular vesicles in colorectal cancer stem cells. Cell Cycle. 2016;15(20):2697–703. 27580100 10.1080/15384101.2016.1218101 PMC5053571

[18] Dai W, Jin X, Han L, Huang H, Ji Z, Xu X, et al. Exosomal lncRNA DOCK9-AS2 derived from cancer stem cell-like cells activated Wnt/β-catenin pathway to aggravate stemness, proliferation, migration, and invasion in papillary thyroid carcinoma. Cell Death Dis. 2020;11(9):743. 32917852 10.1038/s41419-020-02827-w PMC7486896

[19] Han T, Chen L, Li K, Hu Q, Zhang Y, You X, et al. Significant CircRNAs in liver cancer stem cell exosomes: mediator of malignant propagation in liver cancer? Mol Cancer. 2023;22(1):197. 38053070 10.1186/s12943-023-01891-y PMC10696692

[20] Siemann DW, Horsman MR. Modulation of the tumor vasculature and oxygenation to improve therapy. Pharmacol Ther. 2015;153:107–24. 26073310 10.1016/j.pharmthera.2015.06.006 PMC4526350

[21] Geindreau M, Bruchard M, Vegran F. Role of cytokines and chemokines in Angiogenesis in a Tumor Context. Cancers (Basel). 2022;14(10). 10.3390/cancers14102446 PMC9139472 35626056

[22] Collet G, El Hafny-Rahbi B, Nadim M, Tejchman A, Klimkiewicz K, Kieda C. Hypoxia-shaped vascular niche for cancer stem cells. Contemp Oncol (Pozn). 2015;19(1a):A39–43. 25691820 10.5114/wo.2014.47130 PMC4322528

[23] Wang ZF, Liao F, Wu H, Dai J. Glioma stem cells-derived exosomal miR-26a promotes angiogenesis of microvessel endothelial cells in glioma. J Exp Clin Cancer Res. 2019;38(1):201. 31101062 10.1186/s13046-019-1181-4 PMC6525364

[24] Sun X, Ma X, Wang J, Zhao Y, Wang Y, Bihl JC, et al. Glioma stem cells-derived exosomes promote the angiogenic ability of endothelial cells through miR-21/VEGF signal. Oncotarget. 2017;8(22):36137–48. 28410224 10.18632/oncotarget.16661 PMC5482644

[25] Treps L, Perret R, Edmond S, Ricard D, Gavard J. Glioblastoma stem-like cells secrete the pro-angiogenic VEGF-A factor in extracellular vesicles. J Extracell Vesicles. 2017;6(1):1359479. 28815003 10.1080/20013078.2017.1359479 PMC5549846

[26] Lindoso RS, Collino F, Camussi G. Extracellular vesicles derived from renal cancer stem cells induce a pro-tumorigenic phenotype in mesenchymal stromal cells. Oncotarget. 2015;6(10):7959–69. 25797265 10.18632/oncotarget.3503 PMC4480728

[27] Alfaro C, Sanmamed MF, Rodríguez-Ruiz ME, Teijeira Á, Oñate C, González Á, et al. Interleukin-8 in cancer pathogenesis, treatment and follow-up. Cancer Treat Rev. 2017;60:24–31. 28866366 10.1016/j.ctrv.2017.08.004

[28] Kumari A, Kashyap D, Garg VK. Osteopontin in cancer. Adv Clin Chem. 2024;118:87–110. 38280808 10.1016/bs.acc.2023.11.002

[29] Khalil A, Medfai H, Poelvoorde P, Kazan MF, Delporte C, Van Antwerpen P, et al. Myeloperoxidase promotes tube formation, triggers ERK1/2 and akt pathways and is expressed endogenously in endothelial cells. Arch Biochem Biophys. 2018;654:55–69. 30016634 10.1016/j.abb.2018.07.011

[30] Peitzsch C, Tyutyunnykova A, Pantel K, Dubrovska A. Cancer stem cells: the root of tumor recurrence and metastases. Semin Cancer Biol. 2017;44:10–24. 28257956 10.1016/j.semcancer.2017.02.011

[31] Zhang J, Hu Z, Horta CA, Yang J. Regulation of epithelial-mesenchymal transition by tumor microenvironmental signals and its implication in cancer therapeutics. Semin Cancer Biol. 2023;88:46–66. 36521737 10.1016/j.semcancer.2022.12.002 PMC10237282

[32] Fontana R, Mestre-Farrera A, Yang J. Update on epithelial-mesenchymal plasticity in Cancer Progression. Annu Rev Pathol. 2024;19:133–56. 37758242 10.1146/annurev-pathmechdis-051222-122423 PMC10872224

[33] Khan AQ, Hasan A, Mir SS, Rashid K, Uddin S, Steinhoff M. Exploiting transcription factors to target EMT and cancer stem cells for tumor modulation and therapy. Semin Cancer Biol. 2024;100:1–16. 38503384 10.1016/j.semcancer.2024.03.002

[34] Hardin H, Helein H, Meyer K, Robertson S, Zhang R, Zhong W, et al. Thyroid cancer stem-like cell exosomes: regulation of EMT via transfer of lncRNAs. Lab Invest. 2018;98(9):1133–42. 29967342 10.1038/s41374-018-0065-0 PMC6138523

[35] Alzahrani FA, El-Magd MA, Abdelfattah-Hassan A, Saleh AA, Saadeldin IM, El-Shetry ES, et al. Potential effect of Exosomes Derived from Cancer Stem cells and MSCs on progression of DEN-Induced HCC in rats. Stem Cells Int. 2018;2018:8058979. 30224923 10.1155/2018/8058979 PMC6129855

[36] Wu Q, He Y, Liu X, Luo F, Jiang Y, Xiang M, et al. Cancer stem cell-like cells-derived exosomal lncRNA CDKN2B-AS1 promotes biological characteristics in thyroid cancer via miR-122-5p/P4HA1 axis. Regen Ther. 2023;22:19–29. 36582605 10.1016/j.reth.2022.11.005 PMC9772501

[37] Wang L, He J, Hu H, Tu L, Sun Z, Liu Y, et al. Lung CSC-derived exosomal mir-210-3p contributes to a pro-metastatic phenotype in lung cancer by targeting FGFRL1. J Cell Mol Med. 2020;24(11):6324–39. 32396269 10.1111/jcmm.15274 PMC7294132

[38] Wang L, Yang G, Zhao D, Wang J, Bai Y, Peng Q, et al. CD103-positive CSC exosome promotes EMT of clear cell renal cell carcinoma: role of remote MiR-19b-3p. Mol Cancer. 2019;18(1):86. 30975145 10.1186/s12943-019-0997-z PMC6458839

[39] Liu D, Li X, Zeng B, Zhao Q, Chen H, Zhang Y, et al. Exosomal microRNA-4535 of Melanoma Stem cells promotes metastasis by inhibiting Autophagy Pathway. Stem Cell Rev Rep. 2023;19(1):155–69. 35296991 10.1007/s12015-022-10358-4

[40] Li X, Liu D, Chen H, Zeng B, Zhao Q, Zhang Y, et al. Melanoma stem cells promote metastasis via exosomal miR-1268a inactivation of autophagy. Biol Res. 2022;55(1):29. 36182945 10.1186/s40659-022-00397-z PMC9526915

[41] Zhu Y, Huang S, Chen S, Chen J, Wang Z, Wang Y, et al. SOX2 promotes chemoresistance, cancer stem cells properties, and epithelial-mesenchymal transition by β-catenin and Beclin1/autophagy signaling in colorectal cancer. Cell Death Dis. 2021;12(5):449. 33953166 10.1038/s41419-021-03733-5 PMC8100126

[42] Patnam S, Majumder B, Joshi P, Singh AD, Nagalla B, Kumar D, et al. Differential expression of SRY-Related HMG-Box transcription factor 2, Oligodendrocyte Lineage Transcription Factor 2, and Zinc Finger E-Box binding Homeobox 1 in serum-derived extracellular vesicles: implications for Mithramycin Sensitivity and targeted therapy in high-Grade Glioma. ACS Pharmacol Transl Sci. 2024;7(1):137–49. 38230292 10.1021/acsptsci.3c00198 PMC10789128

[43] Shi L, Li B, Zhang Y, Chen Y, Tan J, Chen Y, et al. Exosomal lncRNA Mir100hg derived from cancer stem cells enhance glycolysis and promote metastasis of lung adenocarcinoma through mircroRNA-15a-5p/31-5p. Cell Commun Signal. 2023;21(1):248. 37735657 10.1186/s12964-023-01281-3 PMC10512609

[44] Ji W, Bai J, Ke Y. Exosomal ZFPM2-AS1 contributes to tumorigenesis, metastasis, stemness, macrophage polarization, and infiltration in hepatocellular carcinoma through PKM mediated glycolysis. Environ Toxicol. 2023;38(6):1332–46. 36880413 10.1002/tox.23767

[45] Hwang WL, Lan HY, Cheng WC, Huang SC, Yang MH. Tumor stem-like cell-derived exosomal RNAs prime neutrophils for facilitating tumorigenesis of colon cancer. J Hematol Oncol. 2019;12(1):10. 30683126 10.1186/s13045-019-0699-4 PMC6347849

[46] Gonzalez-Callejo P, Guo Z, Ziglari T, Claudio NM, Nguyen KH, Oshimori N, et al. Cancer stem cell-derived extracellular vesicles preferentially target MHC-II-macrophages and PD1 + T cells in the tumor microenvironment. PLoS ONE. 2023;18(2):e0279400. 36735677 10.1371/journal.pone.0279400 PMC9897575

[47] Ning J, Hou X, Hao J, Zhang W, Shi Y, Huang Y, et al. METTL3 inhibition induced by M2 macrophage-derived extracellular vesicles drives anti-PD-1 therapy resistance via M6A-CD70-mediated immune suppression in thyroid cancer. Cell Death Differ. 2023;30(10):2265–79. 37648786 10.1038/s41418-023-01217-x PMC10589295

[48] Huang S, Zhang P, Yin N, Xu Z, Liu X, Wu A, et al. Glioblastoma stem cell-derived exosomal miR-374b-3p promotes tumor angiogenesis and progression through inducing M2 macrophages polarization. iScience. 2024;27(3):109270. 38487014 10.1016/j.isci.2024.109270 PMC10937837

[49] Bied M, Ho WW, Ginhoux F, Blériot C. Roles of macrophages in tumor development: a spatiotemporal perspective. Cell Mol Immunol. 2023;20(9):983–92. 37429944 10.1038/s41423-023-01061-6 PMC10468537

[50] Chen S, Saeed A, Liu Q, Jiang Q, Xu H, Xiao GG, et al. Macrophages in immunoregulation and therapeutics. Signal Transduct Target Ther. 2023;8(1):207. 37211559 10.1038/s41392-023-01452-1 PMC10200802

[51] Yuan P, Xu X, Hu D, Chen Y, Fan J, Yao S, et al. Highly sensitive imaging of Tumor Metastasis based on the Targeting and polarization of M2-like macrophages. J Am Chem Soc. 2023;145(14):7941–51. 36987634 10.1021/jacs.2c13218

[52] Rouzbahani E, Majidpoor J, Najafi S, Mortezaee K. Cancer stem cells in immunoregulation and bypassing anti-checkpoint therapy. Biomed Pharmacother. 2022;156:113906. 36306594 10.1016/j.biopha.2022.113906

[53] Gabrusiewicz K, Li X, Wei J, Hashimoto Y, Marisetty AL, Ott M, et al. Glioblastoma stem cell-derived exosomes induce M2 macrophages and PD-L1 expression on human monocytes. Oncoimmunology. 2018;7(4):e1412909. 29632728 10.1080/2162402X.2017.1412909 PMC5889290

[54] Wu L, Ye S, Yao Y, Zhang C, Liu W. Oral Cancer stem cell-derived small extracellular vesicles promote M2 macrophage polarization and suppress CD4(+) T-Cell activity by transferring UCA1 and targeting LAMC2. Stem Cells Int. 2022;2022:5817684. 36483681 10.1155/2022/5817684 PMC9723417

[55] Pan Y, Yu Y, Wang X, Zhang T. Tumor-Associated macrophages in Tumor Immunity. Front Immunol. 2020;11:583084. 33365025 10.3389/fimmu.2020.583084 PMC7751482

[56] Yuan Y, Wang L, Ge D, Tan L, Cao B, Fan H, et al. Exosomal O-GlcNAc transferase from esophageal carcinoma stem cell promotes cancer immunosuppression through up-regulation of PD-1 in CD8(+) T cells. Cancer Lett. 2021;500:98–106. 33307156 10.1016/j.canlet.2020.12.012

[57] Cheng WC, Liao TT, Lin CC, Yuan LE, Lan HY, Lin HH, et al. RAB27B-activated secretion of stem-like tumor exosomes delivers the biomarker microRNA-146a-5p, which promotes tumorigenesis and associates with an immunosuppressive tumor microenvironment in colorectal cancer. Int J Cancer. 2019;145(8):2209–24. 30980673 10.1002/ijc.32338

[58] Mirzaei R, Sarkar S, Dzikowski L, Rawji KS, Khan L, Faissner A, et al. Brain tumor-initiating cells export tenascin-C associated with exosomes to suppress T cell activity. Oncoimmunology. 2018;7(10):e1478647. 30288344 10.1080/2162402X.2018.1478647 PMC6169571

[59] Naseri M, Zöller M, Hadjati J, Ghods R, Ranaei Pirmardan E, Kiani J, et al. Dendritic cells loaded with exosomes derived from cancer stem cell-enriched spheroids as a potential immunotherapeutic option. J Cell Mol Med. 2021;25(7):3312–26. 33634564 10.1111/jcmm.16401 PMC8034455

[60] Song H, Liu D, Dong S, Zeng L, Wu Z, Zhao P, et al. Epitranscriptomics and epiproteomics in cancer drug resistance: therapeutic implications. Signal Transduct Target Ther. 2020;5(1):193. 32900991 10.1038/s41392-020-00300-w PMC7479143

[61] Yang Z, Zhao N, Cui J, Wu H, Xiong J, Peng T. Exosomes derived from cancer stem cells of gemcitabine-resistant pancreatic cancer cells enhance drug resistance by delivering miR-210. Cell Oncol (Dordr). 2020;43(1):123–36. 31713003 10.1007/s13402-019-00476-6 PMC12990725

[62] Santos JC, Lima NDS, Sarian LO, Matheu A, Ribeiro ML, Derchain SFM. Exosome-mediated breast cancer chemoresistance via miR-155 transfer. Sci Rep. 2018;8(1):829. 29339789 10.1038/s41598-018-19339-5 PMC5770414

[63] Yang J, Sun G, Hu Y, Yang J, Shi Y, Liu H, et al. Extracellular vesicle lncRNA Metastasis-Associated Lung Adenocarcinoma transcript 1 released from glioma stem cells modulates the inflammatory response of Microglia after Lipopolysaccharide Stimulation through regulating miR-129-5p/High mobility Group Box-1 protein Axis. Front Immunol. 2019;10:3161. 32117213 10.3389/fimmu.2019.03161 PMC7020807

[64] Guo Z, Guo A, Zhou C. Breast Cancer stem cell-derived ANXA6-Containing exosomes sustain Paclitaxel Resistance and Cancer aggressiveness in breast Cancer. Front Cell Dev Biol. 2021;9:718721. 34676207 10.3389/fcell.2021.718721 PMC8523856

[65] Yao W, Guo P, Mu Q, Wang Y. Exosome-derived Circ-PVT1 contributes to Cisplatin Resistance by regulating Autophagy, Invasion, and apoptosis Via miR-30a-5p/YAP1 Axis in Gastric Cancer cells. Cancer Biother Radiopharm. 2021;36(4):347–59. 32799541 10.1089/cbr.2020.3578

[66] Lu T, Li Z, Yang Y, Ji W, Yu Y, Niu X, et al. The Hippo/YAP1 pathway interacts with FGFR1 signaling to maintain stemness in lung cancer. Cancer Lett. 2018;423:36–46. 29452146 10.1016/j.canlet.2018.02.015

[67] Bugter JM, Fenderico N, Maurice MM. Mutations and mechanisms of WNT pathway tumour suppressors in cancer. Nat Rev Cancer. 2021;21(1):5–21. 33097916 10.1038/s41568-020-00307-z

[68] Zhou B, Lin W, Long Y, Yang Y, Zhang H, Wu K, et al. Notch signaling pathway: architecture, disease, and therapeutics. Signal Transduct Target Ther. 2022;7(1):95. 35332121 10.1038/s41392-022-00934-y PMC8948217

[69] Jiang J. Hedgehog signaling mechanism and role in cancer. Semin Cancer Biol. 2022;85:107–22. 33836254 10.1016/j.semcancer.2021.04.003 PMC8492792

[70] Sadrkhanloo M, Entezari M, Orouei S, Ghollasi M, Fathi N, Rezaei S, et al. STAT3-EMT axis in tumors: modulation of cancer metastasis, stemness and therapy response. Pharmacol Res. 2022;182:106311. 35716914 10.1016/j.phrs.2022.106311

[71] Yang Q, Zhao S, Shi Z, Cao L, Liu J, Pan T, et al. Chemotherapy-elicited exosomal miR-378a-3p and miR-378d promote breast cancer stemness and chemoresistance via the activation of EZH2/STAT3 signaling. J Exp Clin Cancer Res. 2021;40(1):120. 33823894 10.1186/s13046-021-01901-1 PMC8022546

[72] Huang YH, Wang WL, Wang PH, Lee HT, Chang WW. EXOSC5 maintains cancer stem cell activity in endometrial cancer by regulating the NTN4/integrin β1 signalling axis. Int J Biol Sci. 2024;20(1):265–79. 38164180 10.7150/ijbs.86275 PMC10750274

[73] Chung WM, Molony RD, Lee YF. Non-stem bladder cancer cell-derived extracellular vesicles promote cancer stem cell survival in response to chemotherapy. Stem Cell Res Ther. 2021;12(1):533. 34627375 10.1186/s13287-021-02600-6 PMC8502272

[74] Chhabra Y, Weeraratna AT. Fibroblasts in cancer: Unity in heterogeneity. Cell. 2023;186(8):1580–609. 37059066 10.1016/j.cell.2023.03.016 PMC11422789

[75] Luo H, Xia X, Huang LB, An H, Cao M, Kim GD, et al. Pan-cancer single-cell analysis reveals the heterogeneity and plasticity of cancer-associated fibroblasts in the tumor microenvironment. Nat Commun. 2022;13(1):6619. 36333338 10.1038/s41467-022-34395-2 PMC9636408

[76] Zhuang J, Shen L, Li M, Sun J, Hao J, Li J, et al. Cancer-Associated fibroblast-derived miR-146a-5p generates a Niche that promotes bladder Cancer Stemness and Chemoresistance. Cancer Res. 2023;83(10):1611–27. 36939397 10.1158/0008-5472.CAN-22-2213

[77] Liu L, Zhang Z, Zhou L, Hu L, Yin C, Qing D, et al. Cancer associated fibroblasts-derived exosomes contribute to radioresistance through promoting colorectal cancer stem cells phenotype. Exp Cell Res. 2020;391(2):111956. 32169425 10.1016/j.yexcr.2020.111956

[78] Hasegawa T, Yashiro M, Nishii T, Matsuoka J, Fuyuhiro Y, Morisaki T, et al. Cancer-associated fibroblasts might sustain the stemness of scirrhous gastric cancer cells via transforming growth factor-β signaling. Int J Cancer. 2014;134(8):1785–95. 24155219 10.1002/ijc.28520

[79] Ren J, Ding L, Zhang D, Shi G, Xu Q, Shen S, et al. Carcinoma-associated fibroblasts promote the stemness and chemoresistance of colorectal cancer by transferring exosomal lncRNA H19. Theranostics. 2018;8(14):3932–48. 30083271 10.7150/thno.25541 PMC6071523

[80] Owusu BY, Galemmo R, Janetka J, Klampfer L. Hepatocyte Growth Factor, a key tumor-promoting factor in the Tumor Microenvironment. Cancers (Basel). 2017;9(4). 10.3390/cancers9040035 PMC5406710 28420162

[81] Hu JL, Wang W, Lan XL, Zeng ZC, Liang YS, Yan YR, et al. CAFs secreted exosomes promote metastasis and chemotherapy resistance by enhancing cell stemness and epithelial-mesenchymal transition in colorectal cancer. Mol Cancer. 2019;18(1):91. 31064356 10.1186/s12943-019-1019-x PMC6503554

[82] Heenatigala Palliyage G, Samart P, Bobbala S, Rojanasakul LW, Coyle J, Martin K, et al. Chemotherapy-induced PDL-1 expression in cancer-associated fibroblasts promotes chemoresistance in NSCLC. Lung Cancer. 2023;181:107258. 37245409 10.1016/j.lungcan.2023.107258 PMC10330668

[83] Li Y, Wang R, Xiong S, Wang X, Zhao Z, Bai S, et al. Cancer-associated fibroblasts promote the stemness of CD24(+) liver cells via paracrine signaling. J Mol Med (Berl). 2019;97(2):243–55. 30564864 10.1007/s00109-018-1731-9

[84] Liu Y, Hua F, Zhan Y, Yang Y, Xie J, Cheng Y, et al. Carcinoma associated fibroblasts small extracellular vesicles with low miR-7641 promotes breast cancer stemness and glycolysis by HIF-1α. Cell Death Discov. 2021;7(1):176. 34238918 10.1038/s41420-021-00524-x PMC8266840

[85] Zhan Y, Du J, Min Z, Ma L, Zhang W, Zhu W, et al. Carcinoma-associated fibroblasts derived exosomes modulate breast cancer cell stemness through exonic circHIF1A by mir-580-5p in hypoxic stress. Cell Death Discov. 2021;7(1):141. 34120145 10.1038/s41420-021-00506-z PMC8197761

[86] Timaner M, Tsai KK, Shaked Y. The multifaceted role of mesenchymal stem cells in cancer. Semin Cancer Biol. 2020;60:225–37. 31212021 10.1016/j.semcancer.2019.06.003

[87] Lan T, Luo M, Wei X. Mesenchymal stem/stromal cells in cancer therapy. J Hematol Oncol. 2021;14(1):195. 34789315 10.1186/s13045-021-01208-w PMC8596342

[88] Lyu T, Wang Y, Li D, Yang H, Qin B, Zhang W, et al. Exosomes from BM-MSCs promote acute myeloid leukemia cell proliferation, invasion and chemoresistance via upregulation of S100A4. Exp Hematol Oncol. 2021;10(1):24. 33789743 10.1186/s40164-021-00220-7 PMC8011411

[89] Hu Y, Liu H, Xiao X, Yu Q, Deng R, Hua L et al. Bone marrow mesenchymal stem cell-derived Exosomes Inhibit Triple-negative breast Cancer Cell Stemness and Metastasis via an ALKBH5-Dependent mechanism. Cancers (Basel). 2022;14(24). 10.3390/cancers14246059 PMC9776833 36551544

[90] Gu H, Yan C, Wan H, Wu L, Liu J, Zhu Z, et al. Mesenchymal stem cell-derived exosomes block malignant behaviors of hepatocellular carcinoma stem cells through a lncRNA C5orf66-AS1/microRNA-127-3p/DUSP1/ERK axis. Hum Cell. 2021;34(6):1812–29. 34431063 10.1007/s13577-021-00599-9

[91] Gao X, Zhou J, Wang J, Dong X, Chang Y, Jin Y. Mechanism of exosomal miR-155 derived from bone marrow mesenchymal stem cells on stemness maintenance and drug resistance in myeloma cells. J Orthop Surg Res. 2021;16(1):637. 34689803 10.1186/s13018-021-02793-9 PMC8543846

[92] Li H, Li F. Exosomes from BM-MSCs increase the population of CSCs via transfer of miR-142-3p. Br J Cancer. 2018;119(6):744–55. 30220706 10.1038/s41416-018-0254-z PMC6173771

[93] Yao X, Mao Y, Wu D, Zhu Y, Lu J, Huang Y, et al. Exosomal circ_0030167 derived from BM-MSCs inhibits the invasion, migration, proliferation and stemness of pancreatic cancer cells by sponging mir-338-5p and targeting the Wif1/Wnt8/β-catenin axis. Cancer Lett. 2021;512:38–50. 33971282 10.1016/j.canlet.2021.04.030

[94] You B, Jin C, Zhang J, Xu M, Xu W, Sun Z, et al. MSC-Derived Extracellular vesicle-delivered L-PGDS inhibit gastric Cancer progression by suppressing Cancer Cell Stemness and STAT3 phosphorylation. Stem Cells Int. 2022;2022:9668239. 35087591 10.1155/2022/9668239 PMC8789473

[95] Lin Z, Wu Y, Xu Y, Li G, Li Z, Liu T. Mesenchymal stem cell-derived exosomes in cancer therapy resistance: recent advances and therapeutic potential. Mol Cancer. 2022;21(1):179. 36100944 10.1186/s12943-022-01650-5 PMC9468526

[96] Li W, Xin X, Li X, Geng J, Sun Y. Exosomes secreted by M2 macrophages promote cancer stemness of hepatocellular carcinoma via the miR-27a-3p/TXNIP pathways. Int Immunopharmacol. 2021;101(Pt A):107585. 34601333 10.1016/j.intimp.2021.107585

[97] Chang J, Li H, Zhu Z, Mei P, Hu W, Xiong X, et al. microRNA-21-5p from M2 macrophage-derived extracellular vesicles promotes the differentiation and activity of pancreatic cancer stem cells by mediating KLF3. Cell Biol Toxicol. 2022;38(4):577–90. 33728488 10.1007/s10565-021-09597-x PMC9343318

[98] Li K, Shi H, Zhang B, Ou X, Ma Q, Chen Y, et al. Myeloid-derived suppressor cells as immunosuppressive regulators and therapeutic targets in cancer. Signal Transduct Target Ther. 2021;6(1):362. 34620838 10.1038/s41392-021-00670-9 PMC8497485

[99] Wang Y, Yin K, Tian J, Xia X, Ma J, Tang X, et al. Granulocytic myeloid-derived suppressor cells promote the stemness of Colorectal Cancer cells through Exosomal S100A9. Adv Sci (Weinh). 2019;6(18):1901278. 31559140 10.1002/advs.201901278 PMC6755519

[100] Peng D, Tanikawa T, Li W, Zhao L, Vatan L, Szeliga W, et al. Myeloid-derived suppressor cells endow stem-like qualities to breast Cancer cells through IL6/STAT3 and NO/NOTCH cross-talk signaling. Cancer Res. 2016;76(11):3156–65. 27197152 10.1158/0008-5472.CAN-15-2528 PMC4891237

[101] Babadag S, Altundag-Erdogan Ö, Akkaya-Ulum YZ, Çelebi-Saltik B. Evaluation of Tumorigenic properties of MDA-MB-231 Cancer stem cells cocultured with Telocytes and Telocyte-Derived Mitochondria following miR-146a inhibition. DNA Cell Biol. 2024. 10.1089/dna.2024.0031 38634821

[102] Rigg E, Wang J, Xue Z, Lunavat TR, Liu G, Hoang T, et al. Inhibition of extracellular vesicle-derived miR-146a-5p decreases progression of melanoma brain metastasis via notch pathway dysregulation in astrocytes. J Extracell Vesicles. 2023;12(10):e12363. 37759347 10.1002/jev2.12363 PMC10533779

[103] Fabbri M. Natural killer cell-derived vesicular miRNAs: a New Anticancer Approach? Cancer Res. 2020;80(1):17–22. 31672842 10.1158/0008-5472.CAN-19-1450 PMC6942618

[104] Lee J, Lee SA, Gu NY, Jeong SY, Byeon JS, Jeong DU, et al. Canine natural killer cell-derived exosomes exhibit Antitumor Activity in a mouse model of Canine Mammary Tumor. Biomed Res Int. 2021;2021:6690704. 34527741 10.1155/2021/6690704 PMC8437631

[105] García-Ortega MB, Aparicio E, Griñán-Lisón C, Jiménez G, López-Ruiz E, Palacios JL et al. Interferon-Alpha decreases Cancer Stem Cell properties and modulates exosomes in Malignant Melanoma. Cancers (Basel). 2023;15(14). 10.3390/cancers15143666 PMC10377490 37509327

[106] Chen J, Xu R, Xia J, Huang J, Su B, Wang S. Aspirin inhibits hypoxia-mediated lung cancer cell stemness and exosome function. Pathol Res Pract. 2019;215(6):152379. 30878308 10.1016/j.prp.2019.03.008

[107] Chen JH, Wu ATH, Bamodu OA, Yadav VK, Chao TY, Tzeng YM et al. Ovatodiolide suppresses oral Cancer Malignancy by Down-regulating Exosomal Mir-21/STAT3/β-Catenin Cargo and Preventing Oncogenic Transformation of normal gingival fibroblasts. Cancers (Basel). 2019;12(1). 10.3390/cancers12010056 PMC7017298 31878245

[108] Li H, Yang C, Shi Y, Zhao L. Exosomes derived from siRNA against GRP78 modified bone-marrow-derived mesenchymal stem cells suppress Sorafenib resistance in hepatocellular carcinoma. J Nanobiotechnol. 2018;16(1):103. 10.1186/s12951-018-0429-z PMC6300915 30572882

[109] Yu L, Gui S, Liu Y, Qiu X, Zhang G, Zhang X, et al. Exosomes derived from microRNA-199a-overexpressing mesenchymal stem cells inhibit glioma progression by down-regulating AGAP2. Aging. 2019;11(15):5300–18. 31386624 10.18632/aging.102092 PMC6710058

[110] Wu H, Mu X, Liu L, Wu H, Hu X, Chen L, et al. Bone marrow mesenchymal stem cells-derived exosomal microRNA-193a reduces cisplatin resistance of non-small cell lung cancer cells via targeting LRRC1. Cell Death Dis. 2020;11(9):801. 32978367 10.1038/s41419-020-02962-4 PMC7519084

[111] Zhao X, Wu X, Qian M, Song Y, Wu D, Zhang W. Knockdown of TGF-β1 expression in human umbilical cord mesenchymal stem cells reverts their exosome-mediated EMT promoting effect on lung cancer cells. Cancer Lett. 2018;428:34–44. 29702191 10.1016/j.canlet.2018.04.026

[112] Zhou W, Zhou Y, Chen X, Ning T, Chen H, Guo Q, et al. Pancreatic cancer-targeting exosomes for enhancing immunotherapy and reprogramming tumor microenvironment. Biomaterials. 2021;268:120546. 33253966 10.1016/j.biomaterials.2020.120546

[113] Sherman MH, Yu RT, Engle DD, Ding N, Atkins AR, Tiriac H, et al. Vitamin D receptor-mediated stromal reprogramming suppresses pancreatitis and enhances pancreatic cancer therapy. Cell. 2014;159(1):80–93. 25259922 10.1016/j.cell.2014.08.007 PMC4177038

[114] Mao X, Xu J, Wang W, Liang C, Hua J, Liu J, et al. Crosstalk between cancer-associated fibroblasts and immune cells in the tumor microenvironment: new findings and future perspectives. Mol Cancer. 2021;20(1):131. 34635121 10.1186/s12943-021-01428-1 PMC8504100

[115] Ghorbaninezhad F, Alemohammad H, Najafzadeh B, Masoumi J, Shadbad MA, Shahpouri M, et al. Dendritic cell-derived exosomes: a new horizon in personalized cancer immunotherapy? Cancer Lett. 2023;562:216168. 37031915 10.1016/j.canlet.2023.216168

[116] Li J, Li J, Peng Y, Du Y, Yang Z, Qi X. Dendritic cell derived exosomes loaded neoantigens for personalized cancer immunotherapies. J Control Release. 2023;353:423–33. 36470333 10.1016/j.jconrel.2022.11.053

[117] Zhou Q, Wei S, Wang H, Li Y, Fan S, Cao Y, et al. T cell-derived exosomes in tumor immune modulation and immunotherapy. Front Immunol. 2023;14:1130033. 37153615 10.3389/fimmu.2023.1130033 PMC10157026

[118] Zhu T, Chen Z, Jiang G, Huang X. Sequential targeting hybrid nanovesicles composed of chimeric Antigen receptor T-Cell-derived exosomes and liposomes for enhanced Cancer Immunochemotherapy. ACS Nano. 2023;17(17):16770–86. 37624742 10.1021/acsnano.3c03456

[119] Si C, Gao J, Ma X. Natural killer cell-derived exosome-based cancer therapy: from biological roles to clinical significance and implications. Mol Cancer. 2024;23(1):134. 38951879 10.1186/s12943-024-02045-4 PMC11218398

[120] Chen Y, Gong L, Cao Y, Liu Z, Wang Y, Cheng H, et al. Reprogramming tumor-associated macrophages by a dually targeted milk exosome system as a potent monotherapy for cancer. J Control Release. 2024;366:395–409. 38184235 10.1016/j.jconrel.2023.12.058

[121] Kim H, Park HJ, Chang HW, Back JH, Lee SJ, Park YE, et al. Exosome-guided direct reprogramming of tumor-associated macrophages from protumorigenic to antitumorigenic to fight cancer. Bioact Mater. 2023;25:527–40. 37056267 10.1016/j.bioactmat.2022.07.021 PMC10087080

[122] Zhang J, Ji C, Zhang H, Shi H, Mao F, Qian H, et al. Engineered neutrophil-derived exosome-like vesicles for targeted cancer therapy. Sci Adv. 2022;8(2):eabj8207. 35020437 10.1126/sciadv.abj8207 PMC8754405

[123] Qiu H, Liang J, Yang G, Xie Z, Wang Z, Wang L, et al. Application of exosomes in tumor immunity: recent progresses. Front Cell Dev Biol. 2024;12:1372847. 38633106 10.3389/fcell.2024.1372847 PMC11021734

[124] He C, Jaffar Ali D, Qi Y, Li Y, Sun B, Liu R, et al. Engineered extracellular vesicles mediated CRISPR-induced deficiency of IQGAP1/FOXM1 reverses sorafenib resistance in HCC by suppressing cancer stem cells. J Nanobiotechnol. 2023;21(1):154. 10.1186/s12951-023-01902-6 PMC10193671 37202772

[125] Ishiguro K, Yan IK, Lewis-Tuffin L, Patel T. Targeting Liver Cancer Stem cells using Engineered Biological nanoparticles for the treatment of Hepatocellular Cancer. Hepatol Commun. 2020;4(2):298–313. 32025612 10.1002/hep4.1462 PMC6996342

[126] Yong T, Zhang X, Bie N, Zhang H, Zhang X, Li F, et al. Tumor exosome-based nanoparticles are efficient drug carriers for chemotherapy. Nat Commun. 2019;10(1):3838. 31444335 10.1038/s41467-019-11718-4 PMC6707218

[127] Li L, Xiong Y, Wang N, Zhu M, Gu Y. Breast Cancer stem cells-derived Extracellular vesicles affect PPARG expression by delivering MicroRNA-197 in breast Cancer cells. Clin Breast Cancer. 2022;22(5):478–90. 35279406 10.1016/j.clbc.2022.02.006

